# Indicators to complement global monitoring of safely managed on-site sanitation to understand health risks

**DOI:** 10.1038/s41545-024-00353-2

**Published:** 2024-07-07

**Authors:** Freya Mills, Tim Foster, Antoinette Kome, Rajeev Munankami, Gabrielle Halcrow, Antony Ndungu, Barbara Evans, Juliet Willetts

**Affiliations:** 1 Institute for Sustainable Futures, University of Technology Sydney, Ultimo, NSW Australia; 2 SNV Netherlands Development Organisation, The Hague, The Netherlands; 3 School of Civil Engineering, University of Leeds, Leeds, UK

**Keywords:** Water resources, Civil engineering

## Abstract

Halfway through the Sustainable Development Goal (SDG) period, there has been little research on the criteria for monitoring safely managed sanitation under SDG target 6.2. For reporting against SDGs, global indicators are necessarily limited and exclude many safety aspects from a public health perspective. Primary survey data from 31,784 households in seven countries in Asia and Africa were analysed, comparing estimates of safely managed on-site sanitation based on global indicators with five complementary indicators of safety: animal access to excreta, groundwater contamination, overdue emptying, entering containments to empty and inadequate protection during emptying. Application of additional criteria reduced the population with safely managed sanitation by 0.4–35% for specific indicators, with the largest impact due to the risk of groundwater contamination, animal access, and containments overdue for emptying. Combining these indicators across the service chain, excluding transport and treatment, found almost three-quarters of on-site systems currently assessed as safely managed with global indicators were considered unsafe based on complementary indicators. A more comprehensive assessment of safety of on-site sanitation can be achieved through these indicators, which could be integrated into national monitoring systems and used to inform sanitation investments that address local health-related risks.

## Introduction

Inadequate sanitation is associated with numerous and varied health risks^1^. There are multiple sources of faecal environmental contamination from inadequate sanitation systems and multiple pathways for exposure^2,3^. The presence of a toilet is therefore an insufficient measure to indicate whether positive health outcomes are likely to be achieved by sanitation improvements^4^, hence numerous authors critiqued the Millennium Development Goal target, expressed solely in terms of access to toilets^5–8^. The Sustainable Development Goal (SDG) target 6.2 of safely managed sanitation services aims to address these limitations by considering the management of excreta from the toilet to final treatment and disposal^9^. The Joint Monitoring Programme (JMP) led the development of global indicators and standardised core questions to enable consistent and practical classification of sanitation services for national and global monitoring (see Table 3)^10^. However, these indicators do not cover all aspects of safety, such as those outlined in WHO guidelines on sanitation and health^1^. The guidelines suggest countries agreeing to the SDG framework should routinely monitor and report on the global indicators, as a minimum, and suggest these are complemented by more nuanced and contextual regional and national indicators. The JMP proposed some expanded indicators, but these focus on expanded definitions of toilet access, for example, privacy of toilet use, and include limited expanded indicators related to the safe management of containments, emptying, conveyance and disposal^10^. Safely managed sanitation as defined for global monitoring, while a significant improvement in monitoring access to improved toilets, should not be assumed to indicate a service level that protects against many key faecal transmission pathways. Since what doesn’t get measured doesn’t get managed^11,12^, relying on global indicators to prioritise investment may result in sanitation improvements that do not address critical health risks.

Despite debate and research on other aspects of SDG 6.2, there has been little assessment of the indicators for safely managed sanitation services nor exploration of the complementary indicators that could address the gaps. Numerous publications have critiqued and suggested improvements to the classification of shared toilets as limited sanitation^13^, the monitoring of progress of lower service levels^14^, the means of implementation targets^15,16^, and explored alternatives for monitoring safely managed water services^17^. However, there has been little discussion on the formation and scope of the indicators for safely managed sanitation services, and even uncertainty about how services will be measured as safely managed^16^. The opinion piece by Rose et al. defined safe sanitation through a communal social lens as based on the ‘social construct that lies at the intersection of knowledge, societal engagement, and controls’^18^. Rose’s paper highlighted the role of the community in monitoring but did not review the indicators for safely managed sanitation or propose alternative indicators relevant to their definition^18^. Beard et al. highlighted the challenges to assessing on-site systems and the need for revised categories for improved sanitation facilities, yet they did not review indicators related to safe management across the service chain^19^. One paper proposed complementary indicators for safely managed sanitation services for national monitoring in Austria^20^. This provided valuable insights for high-income contexts with predominately sewerage services, yet was less applicable for low- and middle-income countries with predominantly on-site sanitation.

National and subnational decision-makers should not rely on global monitoring alone to inform investment. Globally defined indicators for water and sanitation may not adequately capture the national realities and challenges faced by individual countries or best suit the needs of individual countries to assess progress towards national goals^20,21^. Beard et al. argued that for urban sanitation, global monitoring efforts do not provide a clear picture of the challenge of managing excreta at the city scale and that the current indicators have a limited ability to inform policy and action^19^. This paper does not intend to critique the objective and approach of the SDGs or indicators used for global monitoring but to highlight that these indicators are an initial approach to define a ‘safely managed sanitation service’. Indeed, the 2030 Agenda for Sustainable Development recommends that global indicators be complemented by indicators at the regional and national levels, which will be developed by Member States^22^. The Guidelines on Sanitation and Health also suggest more indicators are needed at the utility and sub-national levels to inform local programmes and actions^1^. Although the number of countries able to report against safely managed sanitation has increased, significant data gaps remain, particularly regarding on-site sanitation^23^, making it an opportune time to inform the scope and approach to monitoring sanitation.

Beyond those currently assessed by the global indicators, there are a range of additional exposure pathways associated with inadequate sanitation systems and their management. Animal access to uncovered or inadequately protected faeces can transmit excreta and pathogens to people, surfaces and food, especially in dense settings or places where animals and humans are in close proximity^24–26^. Inadequate subsoil treatment of leachate from unsealed on-site sanitation can contaminate groundwater supplies used for drinking water, with contamination risk influenced by toilet and containment type, soil type, groundwater level and proximity to wells^27^. Poor operation and management of sanitation can also increase exposure to faecal pathogens. Infrequent emptying of on-site sanitation is associated with an increased likelihood of overflowing, malfunction or reduced performance^2^. Infrequent emptying can also lead to unsafe emptying practices, such as entering the pit to remove hardened sludge or informal emptying practices such as wash out, putting both the workers and public at risk of exposure^2,28^. The health risks sanitation workers face during emptying have been increasingly recognised, including direct exposure to faecal pathogens and risks from working in confined spaces^29,30^.

While environmental sampling and detailed health exposure studies and models have improved our understanding of health risks, household surveys can assess potential exposure pathways at a larger scale and lower cost. Several tools, models and detailed research studies have developed methods to investigate critical faecal exposure pathways^25,31–33^. While they have been valuable in demonstrating the high concentration of pathogens in the environment and need to consider multiple exposure pathways, they typically require high skills or equipment and can be difficult to conduct at scale. Household questionnaires, while limited in simple questions and self-reporting, benefit from capturing sanitation data at scale for a relatively low cost when included in broader surveys. Assessment of indicators of pathogen exposure pathways cannot ensure that a system provides 100% protection against human contact with excreta; however, it can point to common failures in sanitation systems that increase the risk of exposure to prioritise improvements or further in-depth investigation. There remains an opportunity to expand household monitoring to better assess and prioritise potential exposure pathways at a larger scale than the field-based exposure assessments.

Recognising that global monitoring is necessarily limited for simplicity and comparability, this paper proposes complementary indicators that could be incorporated into household monitoring to provide a more comprehensive assessment of on-site sanitation focusing on faecal exposure pathways. While research on other aspects of SDG 6.2 led to debate and refinement of indicators (e.g., shared sanitation) for the assessment of safely managed services, as noted above, previous research identified the need for complementary indicators yet did not suggest potential indicators relevant to areas with predominately on-site sanitation, such as is common in low- and middle-income countries. SNV, an international non-government organisation, conducted baseline monitoring between 2018–2019 in 34 urban and rural districts across seven countries to inform and monitor progress of their sanitation programmes. Trained enumerators conducted surveys of 31,784 households, which included global core questions and supplementary questions related to additional exposure pathways as well as qualitative assessments of service provision. The data from health-related household questions were assessed to compare five complementary indicators with the equivalent global sub-indicators for improved, contained and emptied on-site sanitation. This research evaluated the extent to which consideration of critical exposure pathways reduced the proportion of systems classified as safely managed on-site sanitation and analysed the contexts or conditions in which different indicators may be more or less important. This research aims to address the gap in tested complementary indicators relevant to on-site sanitation that could be incorporated into sanitation monitoring systems. The research is timely as national WASH monitoring frameworks continue to be updated to improve reporting against the SDGs, and these relevant complementary indicators to enhance understanding of local health risks and inform sanitation investments.

## Results

**Fig. 1:**
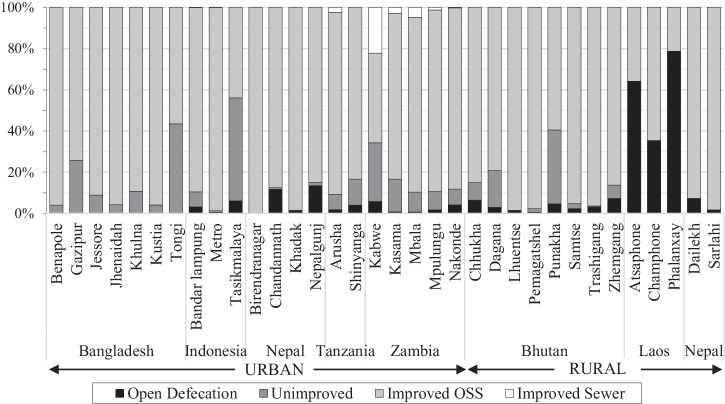
Household access to sanitation by category of facility, as defined by the JMP standard indicator set for 21 urban and 13 rural districts of seven countries based on data collected by SNV in 2018–2019. Complementary indicators were only analysed for the improved sanitation facilities.

As background to the results for the complementary indicators, Fig. 1 presents the overall access to improved sanitation for the 21 urban cities (with populations varying from 21,036–2.67 million) and 13 rural districts (that may include some district centres), see Supplementary Table 1 for details of sample areas. Most households used improved on-site sanitation systems (79% average across countries), which are facilities that aim to hygienically separate excreta from human contact. A small number of households in the cities in Tanzania and Zambia used improved toilets connected to sewers (1%) and on average across countries 10% practised open defecation, predominately in rural Laos. The JMP classifies shared improved toilets as ‘limited sanitation’, which were used by an average of 17% of urban and 6% of rural respondents. This resulted in 65% and 71% of respondents in urban and rural areas reported accessing at least basic sanitation (Supplementary Table 2). While only ‘at least basic’ sanitation can be considered as ‘safely managed’ sanitation services, in this paper the analysis of each indicator considered all improved sanitation facilities, as both shared and private facilities contribute to faecal environmental contamination^34^. The contextual factors included the typology of improved sanitation facilities, of which 89% of households in rural areas reported the use of a pit (i.e., direct pit, off-set pit, two sequential pits, double off-set pit, composting), and 11% reported the use of a tank (septic tank, holding tank, communal septic tank) (see Supplementary Table 2). In urban areas, tanks and pits were equally reported, although this varied between countries. Containments had been in use for an average of 8.6 years in urban areas and 5.8 years in rural areas. Of improved on-site systems, 6% had previously been emptied in rural areas and 22% in urban areas.

### Improved facilities: animal access to excreta

**Fig. 2:**
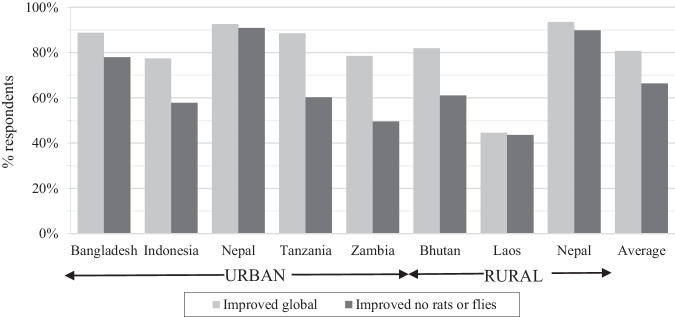
Proportion of households with access to improved sanitation considering the global indicator compared with the complementary indicator that considers toilets that are improved and not accessible to animals. Reduction in improved when considering animals was greatest for Indonesia, Tanzania, Zambia and Bhutan and lowest in Nepal and Tanzania, which also had fewer dry or composting pits.

Moving beyond the high-level assessment of facility type, data was analysed to assess whether facilities classified as improved were still at risk of animal access to excreta, which can result in mechanical transmission of pathogens from animals to humans. The remaining results are presented as country averages for improved clarity in demonstrating the difference between global and complementary indicators, with the results for each of the 34 cities and districts provided in the supplementary materials. On average across all countries 81% of respondents reported using an improved sanitation facility, yet 14% of respondents used improved toilets that were accessible to rats and flies. In urban areas, the proportion of improved facilities reduced by 18% when assessed for animal access, which was a greater reduction than in rural areas (8%). The reduction varied between countries, ranging from 1% in Laos and 2% in urban Nepal to a reduction of 28% and 29% in Tanzania and Zambia, respectively (Fig. 2). The variation between cities or districts within a country was greatest for Bhutan, Bangladesh and Zambia, with the greatest impact (51% reduction) in Zhemgang district, Bhutan. Poorer households and dry toilets had a significantly greater prevalence of animal access than non-poor households or water-based toilets (Table 1).

**Table 1:** Prevalence ratio estimates for the strength of association between context variables and the complementary indicator

Prevalence ratio^a^ [95% CI]		Context variables (household level)						
		Rural/Urban	Poorer households/not poor	GW <5 m / >5 m	Dry/Wet containment	Pit/Tank	Age > 5 yrs/ < 5 yrs	Depth < 3 m / > 3 m
Of improved	Animal access	**0.86** [0.79–0.93]	**1.74** [1.65–1.84]	**1.22** [1.16–1.28]	**4.84** [4.63–5.05]	**2.18** [2.05–2.31]	**0.79** [0.75–0.83]	1.02 [0.95–1.09]
	Uncontained	**0.09** [0.07–0.1]	**1.15** [1.11–1.2]	**0.67** [0.64–0.69]	**0.48** [0.44–0.52]	**0.40** [0.38–0.41]	**1.81** [1.73–1.89]	**0.42** [0.39–0.44]
Of contained (Global)	Ground-water risk	**0.48** [0.46–0.5]	**0.90** [0.87–0.94]	**4.15** [4.01–4.30]	**1.08** [1.04–1.13]	**1.23** [1.19–1.27]	1.00 [0.97–1.02]	**0.50** [0.48–0.52]
Of improved	Emptied (Global)	**0.25** [0.22–0.29]	**1.24** [1.17–1.31]	**1.23** [1.17–1.29]	**0.6** [0.54–0.67]	**1.09** [1.04–1.15]	**5.41** [4.94–5.93]	1.03 [0.96–1.09]
Of improved not emptied	Overdue for emptying	**0.61** [0.57–0.66]	**0.66** [0.62–0.71]	**0.89** [0.85–0.94]	**0.47** [0.42–0.53]	**1.07** [1.02–1.12]	**98.04** [71.9–133.6]	**1.09** [1.04–1.15]
Of improved emptied	Overdue for re-emptying	**2.74** [1.44–5.2]	**0.24** [0.11–0.51]	**0.6** [0.39–0.93]	0.34 [0.08–1.38]	1.14 [0.75–1.71]	1.88 [0.77–4.6]	**3.81** [2.48–5.83]
	Entered	**1.56** [1.18–2.08]	**0.74** [0.61–0.88]	**0.62** [0.53–0.72]	**0.32** [0.19–0.55]	**0.72** [0.63–0.83]	1.03 [0.8–1.34]	1.03 [0.85–1.25]
	Inadequate PPE	**0.77** [0.7–0.86]	**1.12** [1.09–1.15]	0.97 [0.97–1.02]	1.03 [0.98–1.08]	**1.08** [1.05–1.11]	0.97 [0.93–1.01]	0.99 [0.96–1.02]

^a^Significant prevalence ratios are in bold (significance 2-sided *p* > 0.05).

### Containment—Groundwater risk

The assessment of groundwater risk first considers the global indicator for containment, which requires that on-site systems do not discharge excreta to surface environments. The global indicator considers facilities not contained if they overflow or leak waste directly into the surface environment^10,35^. First, we present the findings of the global indicator, as the results for the complementary indicator of groundwater risk are calculated for only those systems classified as contained by the global indicator. Based on the global definition, on average across countries, 66% of respondents used ‘contained’ on-site sanitation and 14% used uncontained systems, made up of 8% with an outlet (i.e., overflow line) to surface environment, 4% having flooded or overflow and 1% with both outlet and overflow. In urban areas, an average 20% of respondents used uncontained systems, with the highest proportion in Bangladesh (57% uncontained), predominately due to outlets to the surface environment (45%) (see Supplementary Fig. 1 and Supplementary Table 2). In rural areas the presence of an outlet was only assessed in Nepal, 1% of improved systems had an outlet, therefore the 4% of respondents using uncontained systems in rural areas was due to issues with flooding and overflow. as SNV’s rural monitoring only assessed the presence of outlets in Nepal and (1% used systems with an outlet to the environment). Factors associated with a significantly greater prevalence of facilities being uncontained were urban areas, wet containments, tanks, deep containments, and systems in deeper groundwater (Table 1). Comparing the different causes, a greater prevalence of flooding and overflow occurred for dry toilets, pits, and poorer households, while a greater prevalence of outlets to surface environment occurred for water flush containments and tanks.

**Fig. 3:**
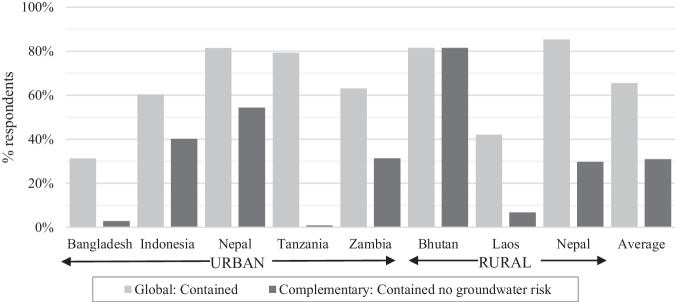
Comparison of global indicator for containment with the complementary indicator that includes on-site systems that are contained and do not pose a high risk of groundwater contamination considering infiltration depth and soil type. The complementary indicator reduced the proportion of systems considered contained across most countries, excluding Bhutan where there is a low risk of groundwater contamination.

While the global indicator assesses releases from on-site sanitation to surface environments, groundwater contamination from on-site sanitation is a critical exposure pathway in some contexts. A risk matrix based on literature was used to assess potential groundwater contamination risk based on household self-reported containment depth and secondary data on groundwater depth and soil type collected for each sub-district or neighbourhood. Methods are described in Table 3, with further details in supplementary materials. The analysis found an average of 35% of the population use systems classified as contained but pose a high risk of contaminating groundwater and ranged from 0% in Bhutan to 78% in Tanzania (see Fig. 3). Most countries had low and high risk areas, indicating the variability of local environmental conditions (see Supplementary Fig. 6 and groundwater depth and soil type in Supplementary Table 1). The exception was Bhutan, where no risk was found in any of the surveyed districts. Recognising that the exposure risk to potentially contaminated groundwater is most relevant when groundwater is used for drinking, further analysis, beyond SNV’s current indicator, considered contamination a high risk only when 25% or more of the respondents in the district reported using groundwater for drinking. Supplementary Fig. 2 presents the adjusted results, which found the proportion of uncontained sanitation due to groundwater risk reduced to an average of 24% considering contamination risks only in areas using groundwater. This revision had the greatest impact in the two cities assessed in Tanzania, with 78% of respondents with contained on-site systems at risk to groundwater reduced to zero since groundwater is not used for drinking. Risks in urban Nepal and Zambia reduced by 4% and 5% respectively, considering some cities had low groundwater use.

### Overdue emptying—unemptied stored in-situ and emptying within the timely threshold

The global indicator for emptying within the assessment of safely managed sanitation considers whether containments were ever emptied. Of all respondents, 10% had improved on-site systems that were previously emptied, 1% built a new pit, 64% were never emptied and 3% didn’t know, which were considered never emptied for analysis (Supplementary Table 2). Emptying rates were lowest in Zambia, Bhutan and Laos (1 to 4%) and highest in Bangladesh (32%) (see Fig. 5). Emptying was more likely for older systems, wet containments and urban areas (Table 1).

**Fig. 4:**
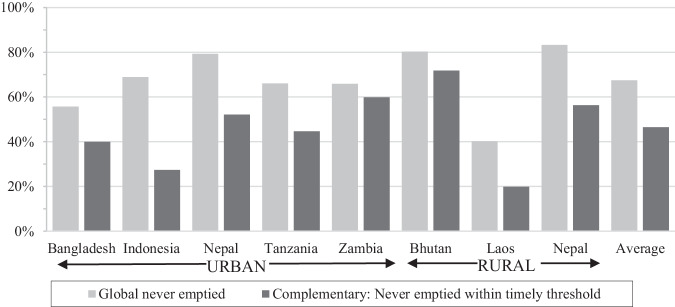
Comparison of the global indicator of households with improved on-site systems that were never emptied with the complementary indicator of improved unemptied systems not overdue for emptying. Contained and not emptied systems are considered safely managed sanitation in global monitoring. However, this complementary indicator demonstrates many of these systems have operated beyond the emptying threshold and are likely full of sludge and at risk of reduced function or overflow.

Many types of containments require regular emptying, so they function as designed or do not overflow. Therefore, the complementary indicator assessed whether unemptied systems were overdue for emptying by comparing years of operation with a calculated timely emptying threshold. The threshold was calculated based on the number of users, containment size and sludge accumulation, estimated for each containment type and each country (see methods in Table 3 and supplementary materials). Compared with the 67% of respondents that used unemptied improved containments, considered by global monitoring as safely stored in-situ, 21% of the population had unemptied improved containments were overdue for emptying (operation years greater than the timely emptying threshold). The largest reductions due to overdue emptying occurred in Indonesia (42%), followed by urban and rural Nepal with 27% reduction, while Zambia was the least impacted by this complementary indicator (6%, see Fig. 4). Within countries, there was some variation between cities or districts, particularly in Nepal where reductions ranged from 11% to 44% between cities. Of improved on-site systems that had never been emptied, urban areas, wet toilets and non-poor households were associated with a significantly greater prevalence of being overdue for emptying, highlighting it is not just an affordability issue (Table 1). Of previously emptied systems, only an average 0.4% of improved on-site systems are overdue for re-emptying, with a maximum reduction of 0.8% of systems in Indonesia (Supplementary Fig. 8 presents disaggregated city and district results).

### Emptying—Occupational health and safety risks

**Fig. 5:**
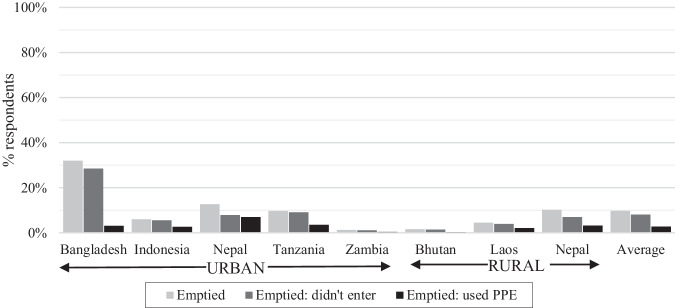
Comparison of ever emptied systems with those emptied following health and safety practices. Much higher rates of emptying occurred in Bangladesh with the complementary indicator of use of PPE having much greater impact than entering in all countries.

While 10% of respondents had improved on-site systems previously emptied, only 8% were emptied without someone entering the containment. From Fig. 5, the greatest reduction in safe emptying when considering entering was in urban Nepal (5% reduction) and Bangladesh (4%), with small decreases in Tanzania and rural Nepal (0–1%), where completely mechanical emptying was more common. Entering was more likely for containments emptied by the household or tenant (24% entered), compared with manual (15%) and mechanical (3%) service providers. Rural areas and wet containments were at greater risk of reported entering to empty, although rural areas were also more likely emptied by users (35%) than urban areas (6%) (Table 1).

The other health and safety indicator was the use of a minimum level of personal protective equipment (PPE), including boots, gloves and a mask. Across all countries, only 3% of respondents used improved on-site sanitation emptied with adequate PPE. The lowest compliance was in Bangladesh where 32% of improved on-site systems had been emptied, yet 29% were systems emptied without minimum PPE. The next largest reduction was in Nepal and Tanzania where 6% of respondents used improved containments emptied without minimum PPE (Fig. 5). Greater PPE compliance was reported for containments emptied by the household than those emptied by service providers and for manual rather than mixed or fully mechanised emptying, noting this data was self-reported. There was slight variation between cities for both indicators, except for Bangladesh only 1–4% of respondents reported systems emptied with adequate PPE despite emptying ranging from 11–44%. The prevalence of inadequate PPE was significantly greater for urban areas and poorer households (Table 1).

### Influence of context variables on the significance of complementary indicators

Analysis of the associations between contextual factors and complementary indicators can inform which indicators or exposure pathways may be most important in specific contexts, recognising that not all indicators may be necessary everywhere. Table 1 indicates which technological, socio-economic and environmental factors were associated with an increased probability of systems failing each indicator. Note that this approach examined factors independently and did not account for the influence of other variables. Compared with rural areas, on-site sanitation systems in urban areas were more likely to be at risk of contaminating groundwater, be overdue for emptying and pose a hazard to workers without adequate PPE. However, the likelihood of workers entering an on-site sanitation system for emptying purposes was greater in rural areas. Compared with median and upper income households, lower-income households were less likely to have systems that were overdue for emptying or be entered by workers for emptying. On-site sanitation systems in areas with shallow groundwater (<5 m) were more likely to pose a contamination risk to groundwater and be accessible to animals, but were less likely to be overdue for emptying or require workers to enter them to empty. Compared with flush or wet containments and tanks, dry containments and pits were more likely to be accessible to animals and pose a risk to groundwater, yet less likely to be uncontained or be entered to empty. Compared with recently built containments, older containments (>5 years old) were more likely to be uncontained and overdue for emptying but less likely to be accessible to animals, with no significant difference in entering to empty.

### Overall analysis of the difference between global and complementary indicators

Table 2 shows the proportion of households meeting global indicators considering the existing definition used by the JMP for global monitoring of safely managed sanitation (on the left). The columns to the right show the reduction in this proportion when considering additional potential exposure pathways of the complementary indicators, including the overall and country average reduction for each indicator. The complementary indicators resulting in the greatest reduction in the proportion of respondents considered safely managed were the indicator of groundwater risk (35% reduction), followed by unemptied containments overdue for emptying (21%) and animal access (14%). While 10% of households had emptied their on-site system (global indicator), very few of these are overdue for re-emptying, and this indicator had the lowest impact (0.4% reduction). Indicators had varied impacts between countries; for example, in Bhutan, animal access caused the greatest reduction, which may be associated with the high use of dry pits, whereas in Laos, considering animal access had a minor impact, while groundwater risk and overdue for emptying had the largest impact on safety. Within-country variability was lower than between-country variability for most indicators except groundwater risk, which had equally high variability within-countries as between-countries (Supplementary Table 8).

**Table 2:** Proportion of respondents meeting global and complementary indicators (I) and the average reduction in the proportion of the population assessed a safe due to each individual complementary indicators average across all countries and per country (II)

Global and complementary indicators	(I) Total respondents assessed as safe for each indicator^a^	(II) Reduction in the population considered safe due to complementary exposure pathways (Reduction% = Global% – Complementary%)									
		All countries		Urban					Rural		
		Ave	Std Dev	BGD	IDN	NPL	TZA	ZMB	BTN	LAO	NPL
**Improved (Global)**	**81%**										
Improved and no animal access	66%	14%	12%	11%	20%	2%	28%	29%	21%	1%	4%
**Contained (Global)**	**66%**										
Contained and low groundwater risk	31%	35%	24%	28%	20%	27%	78%	32%	0%	35%	56%
**Not emptied (Global)**	**67%**										
Not emptied and not overdue for emptying	46%	21%	11%	16%	42%	27%	21%	6%	8%	20%	27%
**Emptied (Global)**	**10%**										
Emptied, not overdue for re-emptying	9%	0.4%	0.3%	0%	1%	1%	0%	0%	0%	1%	0%
Emptied, didn’t enter pit	8%	2%	2%	4%	0%	5%	1%	0%	0%	1%	3%
Emptied, adequate PPE	3%	7%	9%	29%	3%	6%	6%	1%	1%	2%	7%

^a^Global indicator response rate in bold. Reduction is the difference between the response rate for global indicators minus the repsonse rate for complementary indiactors.

**Fig. 6:**
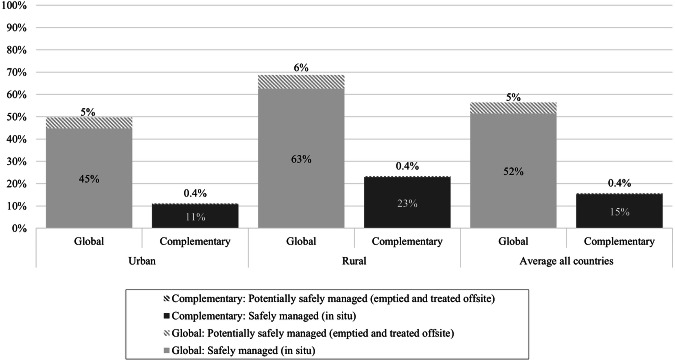
Comparison cumulative estimate safely managed on-site sanitation (excluding transport and treatment) for the global and complementary indicators. Cumulative estimates shown for urban, rural and all respondents and disaggregated by those safely stored in-situ and those safely emptied.

While the indicators were analysed and presented separately to highlight their individual impact and variation between contexts, safely managed sanitation requires cumulative analysis across the service chain as excreta must be managed from containment to treatment. The data allowed for cumulative assessment of safely managed sanitation services considering each household’s response across the service chain (improved, contained, emptied and stored in-situ), recognising that households may fail multiple steps and only achieve safe management if all steps were assessed as safe. A full assessment of safely managed services for systems emptied and disposed off-site was not possible since transport and treatment data was not, and cannot be, collected through household surveys. Therefore the assessment of safely managed sanitation included (a) on-site systems that were contained, not emptied and safely stored in-situ, or (b) emptied and buried in-situ, or (c) potentially safely managed if contained, emptied and removed offsite but with unknown disposal and treatment. Figure 6 shows the results of the combined analysis across the service chain, comparing global indicators with complementary indicators and showing those safely managed by storage in situ (assessment possible with household surveys) and those that are potentially safely managed, assessed up to emptying but not transport and treatment. Considering global indicators, overall 56% of respondents accessed safely managed on-site sanitation services up to emptying, although a proportion of the 5% emptied could be unsafe if not adequately transported and treated. The proportion of households meeting global and complementary indicators was 16%, just over one-quarter of the value found using global indicators only. The difference was larger in urban areas, where the assessment with complementary indicators reduced the proportion of households with safely managed services to just over one fifth of the estimate with global indicators, while in rural areas it was one third. The largest differences were in Bangladesh and Tanzania, where the proportion of households with safely managed services based on global indicators was 26% and 52% respectively compared with 2% and 1% safely managed considering complementary indicators (Supplementary Fig. 3). Laos and rural Nepal had the next largest reductions with the proportion safely managed considering complementary indicators around one tenth of the result using global indicators. Bhutan was the least impacted with complementary indicators resulting in an estimate two-thirds the estimate of safely managed sanitation with global indicators.

## Discussion

While the SDG global indicator 6.2.1a ‘use of safely managed sanitation services’ is an improvement on the monitoring of basic access to toilets, the findings from the analysis of complementary indicators suggest that several faecal exposure risks may remain. There was a stark reduction in the combined estimate of safely managed on-site sanitation from 56% using global indicators to 16% considering five complementary indicators. In all countries, more than one third of systems assessed as safely managed sanitation were considered unsafe based on complementary indicators. Considering the five individual indicators, as these are what can inform where and what to improve across the service chain, the reduction for each complementary indicator compared to the global indicator ranged from 0.4% to 35%. Including these indicators in sanitation monitoring can significantly change whether a sanitation system should be perceived as truly safe, even if it does meet the global criteria for ‘safely managed’. The indicator on groundwater risk had the largest impact, with 35% of systems classified as contained with the global indicator assessed as a high risk for contaminating groundwater. Overdue emptying and animal access had the next greatest impacts, reducing the proportion assessed as safely managed by 22% and 14%, respectively. Given that only 10% of improved systems had ever been emptied, it was not surprising that the complementary indicators on emptying had the lowest overall impact but when considered as a proportion of the emptied systems, these risks remain important. A substantial number of on-site systems failed each indicator to warrant further consideration or uptake of all assessed indicators given the ongoing risks to public health if these exposure pathways are not addressed.

Assessing the individual indicators rather than the overall combined estimate was also important given the variability of risks between and within-countries. In many countries sanitation decisions and investment occur at a sub-national scale, therefore data should be disaggregated to the level needed to inform these decisions^19,36^. Data and risk assessments at a local scale were also emphasised by citywide inclusive sanitation (CWIS) planning and WHO’s sanitation safety planning^37,38^. The impact of the complementary indicators varied both between and within countries which indicated that many risks were context-specific and that global or national assumptions about the priority aspects of safely managed sanitation were unlikely to apply to all sub-national contexts (see disaggregated findings in supplementary materials). Indicators with the greatest between-country variation were groundwater risk, which was high in Tanzania (78% reduction) and zero in Bhutan, and the use of adequate PPE during emptying, which was most impactful in Bangladesh but low in other countries where emptying rates were also low. Within country variation was most evident for groundwater risk, highlighting that decisions on groundwater risk from on-site sanitation are unlikely to be globally or nationally applicable but may be very important in some contexts.

Resources for monitoring sanitation are often limited, therefore this research can inform which contexts specific indicators may be more critical. There remain concerns that monitoring is expensive and diverts funds from already sparse resources for implementation, and debates whether indicators are selected and used to inform decisions^16,21^. Others argue that the limited resources further emphasise the need for careful indicator selection and sufficient data to support decision-making^20,21^. The analysis of prevalence ratios found some contextual factors had prevalence ratios as expected, such as older on-site systems facing a greater prevalence of being overdue for emptying. In contrast, other ratios were less predictable, such as tanks and wet containments associated with a higher prevalence of being entered to empty (rather than externally with a pump) or that poorer households were associated with a lower prevalence of overdue for emptying. Overall, each indicator was found to be important in certain contexts and therefore should be considered for inclusion in local and national monitoring. However, not all indicators will be relevant in every context and the selection of variables should consider whether the context variables or other factors suggest the indicator may be less relevant. For example, if the groundwater is known to be deep in all of Bhutan, the groundwater risk indicator would be unnecessary to monitor or just included in the districts with potentially shallow groundwater. While it may be challenging to decide what indicators will be critical before data collection, this analysis, along with existing background information could be used, or indicator selection could be informed by small pilots or guided by national priorities.

The indicators and methods presented in this paper are not perfect, yet they show a tested way forward to improve monitoring of on-site sanitation that can potentially be integrated into household surveys or routine monitoring systems. Previous research has highlighted the role of development partners in supporting monitoring improvements and national partners through capacity development and data collection, yet noted there was still a lack of tested methods, indicators and recommendations that were directly usable by national governments^16^. National monitoring systems are slow to adopt new indicators and require evidence of testing at scale and their impact on sanitation systems to be receptive to new approaches^39^. Further research could improve complementary indicators, such as refining the indicators on groundwater risk or timely emptying with locally relevant data rather than global assumptions, and further evidence on the relationship between infrequent emptying and groundwater contamination on faecal exposure in different contexts. Other indicators could be included to further understand the cause of the risks, such as why systems are overflowing or being entered to empty, however, there are limits to what can be asked to households as other sources of data may be needed for more technical assessments of containment design and function, or assessment of the availability of mechanical emptying equipment. Research has shown that provision of PPE alone is insufficient to protect public health and also that it is difficult to assess use^30^. Therefore, other indicators for sanitation workers’ health and safety may be selected based on local issues or service objectives, with some examples provided in SNV’s outcome indicators^40^. Lastly, while we discuss health risks, it is also important to recognise that these indicators assess the hazards and there remains limited research on the exposure and illness associated with sanitation related hazards; therefore direct health benefits cannot be guaranteed from achieving these indicators^41^. Nevertheless, investments that address these hazards will progressively reduce pathogens in the environment and contribute towards improved public and environmental health.

The study does not intend to be an exhaustive analysis of all possible indicators for sanitation for different objectives and data sources and instead focuses on a set of recommended household survey questions relevant to identifying and reducing health risks. A limitation of this scope was the exclusion of health risks associated with the transport, treatment, and final disposal, which cannot accurately be assessed from household surveys^42^. The global indicators for transport and treatment are the ‘proportion delivered’ and whether ‘excreta from on-site sanitation receives solid and liquid treatment’. Complementary indicators could also be developed for these steps, for example, public health risks associated with excreta spilled during transport or the actual operation and performance at treatment facilities and the health risks of treatment effluent. Complementary indicators could also be developed to inform other drivers for sanitation investment, such as indicators relevant to environment, finance, equity, service viability, household preferences, etc. Although the sampling presented a diversity of contexts, it was not nationally or globally representative, and testing these indicators in other contexts could confirm their applicability to different settings. Further testing and research on public health risks associated with on-site sanitation could further refine data collection methods and assumptions, particularly locally relevant assumptions for timely emptying and the groundwater risk matrix or to test the sensitivity of the results to different assumptions. Further research could also investigate how other methods of data collection, such as remote sensors, spatial mapping or citizen science, could contribute to, or reduce costs, of montioring sanitation and related health risks^43–45^.

Despite being halfway through the SDG period, there has been little discussion about how this service level of safely managed sanitation is defined and monitored. This paper found that, in many cases, on-site sanitation systems continue to pose a substantial health risk, even if classified as ‘safely managed’ using global indicators and definitions. This is largely because the currently available national data used for global monitoring does not assess all significant exposure pathways. While SDG monitoring created a valuable shift in attention beyond the toilet, national and local monitoring systems need to go beyond the SDG global indicators and integrate additional indicators to enable a more comprehensive assessment of health risks associated with sanitation services. Recognising that household surveys will continue to be a main source of data for on-site sanitation in many low- and middle-income countries, this research suggests that the five indicators analysed can improve the assessment of whether a toilet, on-site sanitation system or emptying practice, poses a hazard to public health. We recommend national and local monitoring systems include these pre-tested indicators to enable a more comprehensive assessment of health risks associated with on-site sanitation. However, indicators should be selected relevant to the context, as not all indicators are relevant everywhere. This paper aims to ignite further debate on the extent to which ‘safely managed sanitation’ is actually safe from a health perspective and that the global definition of safely managed sanitation should not be the uppermost service objective if the ultimate goal is to end human exposure to faecal waste. We recommend further research into whether these or other complementary indicators for safely managed sanitation are critical to assess faecal exposure pathways prevalent in other contexts and to inform further refinements of the proposed data collection and analysis methods. As many countries continue to update monitoring methods to address SDG data gaps, the indicators tested in this paper can be applied immediately in monitoring frameworks and the results can be used to develop even stronger global monitoring systems and inform the post-2030 objectives.

## Methods

Data collection through household surveys was designed and implemented by SNV, a not-for-profit international development organisation that works on water, energy and agriculture in 26 countries in Asia and Africa. This paper draws upon the work of their WASH programmes, where they support local governments to improve sanitation services through urban and rural sanitation and hygiene programmes. These indicators were included in their standardised performance monitoring framework^46^, initially developed in 2010, which also includes other aspects not analysed in this paper, such as off-site sanitation, hygiene and solid waste, and outcomes indicators on service delivery capacities and performance. SNV performance monitoring framework uses ladders for each step of the service chain that combines multiple sub-indicators of functionality, sustainability and risk. This paper presents the sub-indicators separately for clarity and ease of applying the indicators to other monitoring frameworks.

### Data collection

In partnership with local governments, SNV conducted baseline monitoring between 2018 and 2019 in 18 urban and 13 rural districts across seven countries in Asia and Africa. A total of 31,784 households were surveyed, with 26,436 households in urban cities and 5348 in rural districts (that include some district centres). In three Bangladesh cities (Jhenaidah, Khulna and Kushtia), the baseline survey included slightly different indicators; therefore the mid-term data collected in 2019 was used in this analysis for consistency. SNV received approval from each of the individual countries to collect the data and obtained informed consent from all respondents and data was anonymised by SNV for every survey. The University of Technology of Sydney Human Research Ethics Committees conducted an ethical review of the data use and analysis which was approved on 6 July 2021 (UTS HREC ETH20-5620). The standardised survey tools were translated into local languages and implemented with mobile phone-based technology (AKVO Flow). Enumerators were either local government staff or hired enumerators, managed and trained by SNV staff. A multi-stage sampling method was adopted, with the primary sampling unit of wards and districts from the programme locations previously determined by the national government. The proportion method for sample size was used to determine district/ward sample size, assuming a 5% level of significance and 2–3% margin of error. The secondary sampling unit (SSU) was country-specific; for example, in Indonesia it was village (*Kelurahan*), which were randomly selected, and samples were distributed proportionally to the village population. In areas where there were administrative units below the SSU (i.e., neighbourhoods), further random sampling was done and each selected neighbourhood was allocated an equal number of households to be surveyed. Systematic sampling was used to identify the household within each neighbourhood or village. Sample size and details of each city or district are provided in Supplementary Table 1.

### Complementary indicator data collection and analysis

The indicators and data collection approaches were developed for SNV’s global sanitation and hygiene monitoring framework for their multi-year urban and rural sanitation and hygiene programmes. The indicators were selected to go beyond the global indicators (see Table 3), recognising that monitoring smaller incremental changes allowed for greater learning and pathways for sanitation service improvements. SNV assessed 40 complementary impact indicators, including a range of behavioural elements (e.g., functionality, use, maintenance), hygiene, and health and safety, as well as outcome indicators to assess service provision qualitatively. The indicators analysed in this paper were a selection of the most relevant impact indicators to assess health risks along the sanitation service chain from toilet to emptying. The global indicators presented in Table 3 are based on the current approach to monitoring SDG 6.2.1a as explained in the recent progress reports, although it is recognised that many countries do not yet collect data on all of these indicators, particularly containment and emptying. Table 4 presents the data collection methods, predominately household questionnaires but also enumerator observation and secondary data for the groundwater risk assessment. Further details of the analysis of groundwater risk and timely emptying are presented in the supplementary material.

**Table 3:** Comparison of global and complementary indicators and literature justification for indicators selected

JMP global indicators^10^	Complementary indicators	Justification
Improved toilet facilities^a^ include flush/pour flush toilets connected to piped sewer systems, septic tanks or pit latrines; pit latrines with slabs; and composting toilets.	Animal access to excreta: Rats and flies cannot enter and exit the toilet or containment	The JMP indicator of an improved toilet is defined as hygienically separating human excreta from human contact. Although some currently included criteria applied to the assessment of improved sanitation are relevant to animal access, such as slabs on pit latrines and excluding unenclosed faeces such as hanging latrines, these criteria do not directly address fly or vermin access to excreta. The following evidence highlights the importance of the exposure pathway of animal access to excreta. - Insects can transport pathogens from excreta to people, surfaces and food^24,26,47,48^. - Flies have been shown to carry a variety of enteric pathogens, including bacteria and protozoa^49–51^ - Flies can transmit high levels of faecal contamination to exposed food, especially in high-contamination settings such as slums or markets^24^ but also in rural areas^26^. - Flies are abundant in urban areas when unsanitary conditions prevail and frequently contact excrement, especially when they are poorly contained^47^. - Rodents and insects are known vectors of human pathogens and diseases, are are attracted to on-site saniation systems yet literature directly linking human pathogens and outbreaks of diseases transmitted by vectors from pit latrines to humans is still scarce^52,53^.
Contained: On-site sanitation facilities that do not overflow or discharge excreta directly to the surface environment^b^.	Groundwater risk: Low risk to groundwater from subsurface leaching of pits or tanks	Globally, half of the world’s population relies on groundwater for water supply, and half also use on-site systems for sanitation^35^. This combination poses a risk of faecal pathogens contaminating the drinking water of many hundreds of millions of people worldwide^54^. Subsurface infiltration of liquids is a crucial component of most on-site sanitation systems and is the mechanism relied on to treat faecal pathogens. However, in certain conditions due to soil type, groundwater level or hydraulic loading, sanitation systems can contaminate groundwater supplies^1,55^. Recent reports indicate that a high proportion (typically 30-50%) of water from wells contains faecal indicator bacteria, such as E. coli or faecal coliforms^54^. Studies in both high- and low-income countries have shown a link between well contamination and on-site sanitation^56^. Although the mechanism of contamination cannot always solely be attributed to sanitation due to numerous potential local or other contamination pathways^27,57^. Pathogen transport in soil and groundwater varies significantly, with viruses and bacteria found to travel 2-50 m depending on the pathogen and ground conditions^27^. While these variations have made it difficult to set standard limits on siting or use of on-site sanitation in areas of groundwater use^58^, the most commonly reported factors that influence contamination risk are soil type and groundwater depth. A greater risk of contamination is expected for permeable soils such as coarse sand, gravel, and fractured rocks^27^. Groundwater depth is important as saturated soils can reduce pathogen removal and increase transport. Groundwater levels near or above the pit base have been shown to increase the pathogen horizontal travel distances compared to unsaturated conditions^58^ and an adequate infiltration depth (i.e., >2 m) is needed to reduce microbial contaminants to minimal levels^59^.
Disposed in-situ: Improved on-site sanitation facilities that are contained, not emptied and stored on-site. (Also relates to Emptying – see below)	Not emptied OSS within timely threshold: Pits or tanks not emptied and not overdue for emptying Emptied OSS within timely threshold: Pits or tanks have been emptied and not overdue for re-emptying	The global indicator for emptying assesses whether containments have ever been emptied. If contained and emptied, containments can be considered safely managed if excreta are buried in a covered pit on-site or if there is evidence that faecal sludge is delivered to a treatment site and treated. Never emptied containments, if assessed in the previous step as contained, are considered to be safely managed by treatment and disposal in-situ, irrelevant of how long they have been operating. The global indicator focuses on the transport of removed faecal sludge given the evidence that a high proportion is not delivered to treatment^60^. However, it does not consider the varied occupational and environmental health and safety issues associated with infrequent emptying. These include reduced performance of septic tanks operating longer than designed^61^ and high solids in effluent causing clogging of infiltration systems; allowing containments to overflow to surface before emptying^62^; full pit latrines being washed out into drains or floodwaters^19,62^; settled sludge hardening and difficult to remove by mechanical pumps; or emergency emptying when toilets or pits overflow often leading to unsafe emptying practices^63^. Low emptying rates may also indicate where there are inadequate emptying services or low awareness of the need for emptying.
Emptied: Improved on-site sanitation storage facilities with containments (septic tanks or latrines) which have ever been emptied.	Emptying health and safety risks: Emptying of containments does not pose a health and safety risk to workers or the public	Emptying on-site sanitation is an activity that presents many health risks to the sanitation workers involved in emptying, as well as the owners of the containment and surrounding community. A systematic review of the health risks among sanitation workers found evidence of sanitation workers being at an increased risk of gastroenteritis and respiratory conditions and may be at increased risk of musculoskeletal disorders and mental/social health conditions^64^. Studies in India and Africa identified multiple possible safety hazards and workers exposed to various occupational risks, including exposure to faecal pathogens, heavy labour, working in confined spaces, and the use of hazardous chemicals, which could lead to injuries, illnesses, and death^29,30^. While both manual and mechanical emptying are accepted practices in the indicators for SDG 6.2.1 and in the WHO Guidelines for Sanitation and Health, risks were found to be more acute for manual or informal emptiers, with some countries prohibiting manual emptying^1,65^. The guidelines recommend minimising manual emptying where possible and avoid entering pits by transitioning to pumps^1^. Correct and consistent use of PPE was a commonly suggested approach to reduce occupational risks, however, it has been recognised that the use of PPE is a challenge and poor fitting or unsuitable equipment and lack of availability, particularly for informal workers, remains a challenge^29,30^. Many papers emphasised that PPE alone is inadequate to reduce health risks from emptying and regulation, enforcement, finance and behaviour change are needed, as well as more data about sanitation workers needs and challenges^1,29,30^.

^a^Basic sanitation includes improved facilities that are not shared with other households. Indicators on transport and treatment not shown.

^b^The 2018 Core questions document^10^ indicates that outlet to surface and overflow are expanded indicators, however, these are actually core questions in the current methods of monitoring safely managed sanitation based on the latest progress reports^23,35^.

**Table 4:** Methods for data collection and analysis for complementary indicators

Indicators	Question^a^	Method and analysis
Animal access to excreta: Rats and flies cannot enter and exit the toilet or containment	Can rats access the faeces in any way? If not, does the toilet pan or slab allow flies to enter and exit the pit?	Where possible this was observed and if not it was asked to the respondent. Rat access was assessed by observation of the type of pit structure, with hanging latrines and pits without a slab allowing rat access, as well as pits without covers or water seals not funcioning. For fly access, observation of the toilet water seal, pan cover and covering or mesh on vents.
Contained: On-site sanitation facilities that do not overflow or discharge excreta directly to the surface environment.	Is there an effluent outlet? Where does the effluent go? Does the toilet flood at any time of the year? Does the pit or toilet leak, overflow or flood at any time of the year? If so, how often does it leak or overflow?	The global indicator for contained was assessed by two sub-indicators Firstly, the outlet, sometimes referred to as overflow line, was assessed through ‘Is there an effluent outlet?’ and ‘Where does the effluent go?’. Systems were classified as uncontained if there was an effluent outlet discharging to surface environments (i.e., streets, open fields, drains, streams and other waterways).^b^ Secondly overflow or flooding were assessed as whether the toilet flooded at any time and whether there was leaking, overflow or flooding more than once in the last year.^c^ If either or both of these were positive, the system was assessed as uncontained.
Groundwater risk: Low risk to groundwater from subsurface leaching of pits or tanks	Household questionnaire: How deep is the toilet pit below the surface? What is the main water source for drinking in this household? Non-household survey data: What is the predominant soil type? What is the typical depth of groundwater?	As this indicator is considered to go beyond the global indicators, the analysis was only for systems classified as contained based on global indicators. Soil type and groundwater depth for each neighbourhood or sub-district were sourced from secondary data (government maps and databases) and interviews with government environmental staff, well drillers and local leaders. Groundwater risk was assessed based on a risk matrix considering soil type and infiltration depth from the British Geological Society Guidelines for Assessing the Risk to Groundwater from On-Site Sanitation (AGROSS Table 4.3)^66^. This indicates that an infiltration depth less than 5 m is always unsafe, greater than 20 m is always safe, and between 5–20 m is unsafe in coarse sand, gravel and fractured rock, but safe in other soil types. The infiltration depth was calculated as the difference between the groundwater depth from the secondary data (using the upper limit of the range, see Supplementary Table 1) and containment depth from household self-reported depth, limited to a maximum of 10 m as deeper estimates were considered unrealistic (see Supplementary Table 5). 3% of households did not know the depth of their containment, and therefore, this population was excluded from the calculation. If the result of the matrix was high risk, the system was considered not safely contained. The analysis also assessed the proportion of cities or districts using groundwater sources (all types of wells, bores and springs) for drinking water supply, although this was not included in the complementary indicator.
Timely emptying: Unemptied pits or tanks, age below timely emptying threshold.	Where do the faeces go after the toilet (i.e., pit, tank, drain)*?* How old is your toilet (pit/tank)? Has the pit or tank ever been emptied? When was the last time the pit or tank was emptied? (if emptied)	The timely emptying threshold was the calculated number of years of operation after which the containment was expected to be full of sludge and require emptying (or alternatively, the construction of a new pit for pit latrines). Given containments are different sizes and fill up at different rates which vary with context, national estimates of timely emptying thresholds were calculated for different containment categories (single and double pit latrines, single and double composting latrines and septic tanks). The threshold was calculated from existing national data or rapid assessments of the average containment volume, number of users and sludge (blanket) accumulation rates based on literature. For pit latrines estimates of sludge accumulation range from 19–70 L/c/a, with 40 L/c/a suggested for design which was the assumed value for dry or composting toilets^67–69^. For septic tanks and wet pits, data ranged from 13–54 L/c/a and recommended values for design for wet pits of 60 L/c/a and 80 L/c/a for septic tanks based on literature from South Africa and unpublished data from Malaysia wastewater authority sludge emptying programme were adopted for the analysis^67,70–72^. More sludge accumulation data relevant to different containment types and contexts would improve the estimates and national sludge accumulation data would be preferred. For containments that have never been emptied, to be considered safely treated and stored in situ the age of the toilet must be less than the timely emptying threshold, allocated based on country and containment type. For emptied systems, as self-reported by households, the time since previous emptying must be less than the threshold to be considered safely emptied. Unknown emptying responses were classified as never emptied as per global monitoring. Given emptying relies on self-reporting, other methods could be employed to improve the accuracy of this response, but may depend on the context (e.g., receipts of emptying service, regulator or service provider data on emptying rates). Pits that were covered when full and a new one built were considered safe, as per the global indicators. The time emptying thresholds for urban and rural areas are provided in supplementary materials Supplementary Tables 6 and 7.
Timely re-emptying: Years since pits or tanks were emptied within timely emptying threshold		
Emptying health and safety risks: Emptying of containments does not pose a health and safety risk to workers or the public	To empty the pit, did someone need to enter the pit? Did you observe any of the following safety measures during emptying? (use of boots, gloves and a mask)	For containments reported as previously emptied, the first question assessed whether someone entered the pit or tank to empty. This was asked separately from the PPE question due to the high risk of this behaviour. The second question was a multiple-response question, asking whether the respondent observed any of the health and safety practices related to protective equipment, of which all three were required to be considered safe, while a response of some or none was considered unsafe.

^a^The household questions and response categories are provided in supplementary material Supplementary Table 9.

^b^While outlets should be considered for all containments, in SNV’s monitoring framework it was not included in rural areas of Bhutan or Laos as pre-testing indicated this practice did not occur in rural areas.

^c^Note these questions differ slightly from the questions in the JMP Core questions^10^ and the recently included question in UNICEF’s household surveys (MICS7) that assesses releases of excreta to the surface through overflow, floods or containment collapse^73^.

### Data analysis

The objective of the data analysis was to quantify the extent to which the complementary indicators changed the assessment of safely managed sanitation, compared with the current global indicators, as defined by the JMP. The data were first analysed to determine the respondents with at least improved sanitation (as defined in Table 3 and presented in Fig. 1). The complementary indicator analysis was only conducted for households with improved sanitation facilities. While safely managed sanitation is only assessed for basic facilities (improved facilities that are not shared), this would have substantially reduced the complementary indicator analysis from Tanzania and Zambia, where sharing was high, and the health risks assessed are equally relevant to both shared and not shared facilities. The indicators were presented for each step and then combined along the chain until the emptying step, as the safety of transport and treatment cannot be determined from household monitoring, which was the scope of this research. Cumulative assessment was possible for each respondent due to the availability of a single dataset that included multiple indicators, which is often not the case for global monitoring data which typically relies on ratios for cumulative assessment. Good quality data management and analysis is necessary to enable this type of analysis which can also permit disaggregated analysis considering inequalities and gender.

The prevalence ratio of the association between contextual variables and the complementary indicators being safe or unsafe was analysed using SPSS v28.0. The variables (or risk factors) included rural vs. urban, poorer households (lowest two wealth quintiles based on country specific assessment of assets) vs. not poor, groundwater depth less vs. more than 5 m, dry containments vs. wet (pour or cistern flush), pits (all types) vs. tanks (septic, holding tank), toilet age more vs. less than 5 years old, containment depth less vs. greater than 5 m. Associations of prevalence were considered significant if the 2-sided *p*-value was less than 0.05. This analysis does not propose a correlation between indicators and variables since other factors may influence but aims to inform which contexts the indicators may be more critical to monitor.

The results were presented per country and with the overall country average rather than total responses, given that sample sizes varied between countries. Data disaggregated at the city or district level are presented in supplementary materials. References to country findings were representative of the cities or districts assessed (see Supplementary Table 1) and were not nationally representative.

### Supplementary information

Supplementary Materials

## Data Availability

The data that support the findings of this study are available on reasonable request from the corresponding author. The data are not publicly available due to them containing information that could compromise research participant privacy.

## References

[1] WHO. *Guidelines on Sanitation and Health* (World Health Organization, 2018).

[2] Manga M (2022). Public health performance of sanitation technologies in Tamil Nadu, India: initial perspectives based on E. coli release. Int. J. Hyg. Environ. Health.

[3] Robb K (2017). Assessment of fecal exposure pathways in low-income urban neighborhoods in Accra, ghana: rationale, design, methods, and key findings of the SaniPath study. Am. J. Trop. Med. Hyg..

[4] Cumming O (2019). The implications of three major new trials for the effect of water, sanitation and hygiene on childhood diarrhea and stunting: a consensus statement. BMC Med..

[5] Weststrate J, Dijkstra G, Eshuis J, Gianoli A, Rusca M (2019). The sustainable development goal on water and sanitation: learning from the millennium development goals. Soc. Indic. Res..

[6] Satterthwaite D (2016). Missing the Millennium Development Goal targets for water and sanitation in urban areas. Environ. Urban..

[7] Tilley E (2014). Looking beyond technology: an integrated approach to water, sanitation and hygiene in low income countries. Environ. Sci. Technol..

[8] Munamati M, Nhapi I, Misi SN (2015). Monitoring sanitation performance: unpacking the figures on sanitation coverage. J. Water Sanit. Hyg. Dev..

[9] Bain R, Johnston R, Mitis F, Chatterley C, Slaymaker T (2018). Establishing sustainable development goal baselines for household drinking water, sanitation and hygiene services. Water.

[10] UNICEF and WHO. *Core Questions on Water, Sanitation and Hygiene for Household Surveys: 2018 Update*. 1–24 https://washdata.org/monitoring/methods/core-questions (2018).

[11] Barnett P (2015). If what gets measured gets managed, measuring the wrong thing matters. Corp. Financ. Rev..

[12] Essex B, Koop SHA, Van Leeuwen CJ (2020). Proposal for a national blueprint framework to monitor progress on water-related sustainable development goals in Europe. Environ. Manag..

[13] Evans B (2017). Limited services? The role of shared sanitation in the 2030 Agenda for Sustainable Development. J. Water Sanit. Hyg. Dev..

[14] Kempster S, Hueso A (2018). Moving up the ladder: Assessing sanitation progress through a total service gap. Water Switz..

[15] Bartram J, Brocklehurst C, Bradley D, Muller M, Evans B (2018). Policy review of the means of implementation targets and indicators for the sustainable development goal for water and sanitation. Npj Clean. Water.

[16] Guppy L, Mehta P, Qadir M (2019). Sustainable development goal 6: two gaps in the race for indicators. Sustain. Sci..

[17] Charles KJ, Nowicki S, Bartram JK (2020). A framework for monitoring the safety of water services: from measurements to security. Npj Clean Water.

[18] Rose JB, Hofstra N, Murphy HM, Verbyla ME (2019). What is safe sanitation?. J. Env. Eng.

[19] Beard VA, Satterthwaite D, Mitlin D, Du J (2022). Out of sight, out of mind: understanding the sanitation crisis in global South cities. J. Environ. Manag..

[20] Germann V, Langergrabe G (2022). Going beyond global indicators-policy relevant indicators for SDG 6 targets in the context of Austria. Sustain. Sustain. Switz.

[21] Hering JG (2017). Managing the ‘monitoring imperative’ in the context of SDG Target 6.3 on water quality and wastewater. Sustain.

[22] UN General Assembly. *Transforming Our World: The 2030 Agenda for Sustainable Development*. https://documents-dds-ny.un.org/doc/UNDOC/GEN/N15/291/89/PDF/N1529189.pdf?OpenElement (2015).

[23] UNICEF and WHO. *Progress on Household Drinking Water, Sanitation and Hygiene 2000–2022: special focus on gender.* New York: United Nations Children’s Fund (UNICEF) and World Health Organization (WHO) (2023).

[24] Lindeberg YL (2018). Can Escherichia coli fly? The role of flies as transmitters of E. coli to food in an urban slum in Bangladesh. Trop. Med. Int. Health.

[25] Capone D (2023). Urban onsite sanitation upgrades and synanthropic flies in Maputo, Mozambique: effects on enteric pathogen infection risks. Env. Sci. Technol..

[26] Doza S (2018). Prevalence and association of Escherichia coli and diarrheagenic Escherichia coli in stored foods for young children and flies caught in the same households in Rural Bangladesh. Am. J. Trop. Med. Hyg..

[27] Graham JP, Polizzotto ML (2013). Pit latrines and their impacts on groundwater quality: a systematic review. Environ. Health Perspect..

[28] Jenkins MW, Cumming O, Scott B, Cairncross S (2014). Beyond ‘improved’ towards ‘safe and sustainable’ urban sanitation: Assessing the design, management and functionality of sanitation in poor communities of Dar es Salaam, Tanzania. J. Water Sanit. Hyg. Dev..

[29] Gautam M, Wankhade K, Sarangan G, Sudhakar S (2021). Framework for addressing occupational safety of de-sludging operators: a study in two Indian cities. J. Environ. Manag..

[30] Philippe S (2022). Challenges facing sanitation workers in Africa: a four-country study. Water Switz..

[31] Okaali DA (2019). Tools for a comprehensive assessment of public health risks associated with limited sanitation services provision. EPB Urban Anal. City Sci..

[32] Wang Y (2022). Quantitative assessment of exposure to fecal contamination in urban environment across nine cities in low-income and lower-middle-income countries and a city in the United States. Sci. Total Environ..

[33] Gretsch SR (2016). Quantification of exposure to fecal contamination in open drains in four neighborhoods in Accra, Ghana. J. Water Health.

[34] Berendes DM (2018). Urban sanitation coverage and environmental fecal contamination: Links between the household and public environments of Accra, Ghana. PLoS One.

[35] WHO and UNICEF. *Progress on Household Drinking Water, Sanitation and Hygiene 2000-2020: Five Years into the SDGs* (2021).

[36] Quispe-Coica A, Pérez-Foguet A (2022). From the global to the subnational scale: Landing the compositional monitoring of drinking water and sanitation services. Sci. Total Environ..

[37] Schrecongost A, Pedi D, Rosenboom JW, Shrestha R, Ban R (2020). Citywide inclusive sanitation: a public service approach for reaching the urban sanitation SDGs. Front. Environ. Sci.

[38] WHO. *Sanitation Safety Planning*. https://www.who.int/publications/i/item/9789240062887 (2023).

[39] Robinson, A. & Peal, A. *Safely Managed Sanitation in the Global Sanitation Fund*. Geneva: The Sanitation & Hygiene Fund, (2020).

[40] SNV. *SSH4A Performance Monitoring Framework, Part 2. Outcome Indicators*. The Hague: SNV (2019).

[41] Sclar GD (2016). Assessing the impact of sanitation on indicators of fecal exposure along principal transmission pathways: A systematic review. Int. J. Hyg. Environ. Health.

[42] Turman-Bryant N, Clasen TF, Fankhauser K, Thomas EA (2018). Measuring progress towards sanitation and hygiene targets: a critical review of monitoring methodologies and technologies. Waterlines.

[43] Andres L, Boateng K, Borja-Vega C, Thomas E (2018). A review of in-situ and remote sensing technologies to monitor water and sanitation interventions. Water.

[44] Fraisl D (2020). Mapping citizen science contributions to the UN sustainable development goals. Sustain. Sci..

[45] Okaali DA, Hofstra N (2018). Present and future human emissions of Rotavirus and Escherichia coli to Uganda’s surface waters. J. Environ. Qual..

[46] SNV. *SSH4A Performance Monitoring Framework, Part 1. Introduction and Impact Indicators*. The Hague: SNV (2019).

[47] Graczyk TK, Knight R, Gilman RH, Cranfield MR (2001). The role of non-biting flies in the epidemiology of human infectious diseases. Microbes Infect..

[48] Capone D (2021). Impact of an urban sanitation intervention on enteric pathogen detection in soils. Env. Sci. Technol..

[49] Fotedar R (2001). Vector potential of houseflies (Musca domestica) in the transmission of Vibrio cholerae in India. Acta Trop..

[50] Khin Nwe OO, Sebastian AA, Aye T (1989). Carriage of enteric bacterial pathogens by house flies in Yangon, Myanmar. J. Diarrhoeal Dis. Res..

[51] Szostakowska B (2004). Cryptosporidium parvum and giardia lamblia recovered from flies on a cattle farm and in a landfill. Appl. Environ. Microbiol..

[52] Gwenzi W (2023). The pit latrine paradox in low-income settings: a sanitation technology of choice or a pollution hotspot?. Sci. Total Environ..

[53] Nadimpalli ML (2020). Urban informal settlements as hotspots of antimicrobial resistance and the need to curb environmental transmission. Nat. Microbiol..

[54] Ravenscroft, P. & Lytton, L. *Practical Manual on Groundwater Quality Monitoring*. Washington DC: World Bank (2022).

[55] Schmoll O (2013). Protecting groundwater for health: managing the quality of drinking-water sources. Water Intell. Online.

[56] Murphy HM (2020). Septic systems and rainfall influence human fecal marker and indicator organism occurrence in private wells in Southeastern Pennsylvania. Environ. Sci. Technol..

[57] Ravenscroft P (2017). The public health significance of latrines discharging to groundwater used for drinking. Water Res..

[58] Mbae, M., Hansen, P., Way, C., Mills, F., Willetts, J., Foster, T. & Evans, B. Onsite sanitation systems and contamination of groundwater: a systematic review of the evidence for risk using the source-pathway-receptor model. Manuscript submitted for publication (2024).

[59] Lewis, W. J., Foster, S. S. D. & Drasar, B. S. *The Risk of Groundwater Pollution by On-Site Sanitation in Developing Countries*. Dubendorf: International Reference Centre for Wastes Disposal (IRCWD) Report No. 01/82 (1982).

[60] Peal A (2020). Estimating safely managed sanitation in urban areas; lessons learned from a global implementation of excreta-flow diagrams. Front. Environ. Sci.

[61] Tang T (2022). Accelerating progress towards universal water sanitation and hygiene (WASH): governance, technology and data for urban settings. Environ. Plan. B Urban Anal. City Sci..

[62] Williams, A. R. & Overbo, A. *Unsafe Return of Human Excreta to the Environment: A Literature Review*. Chapel Hill: The Water Institute at UNC (2015).

[63] Greene N, Hennessy S, Rogers TW, Tsai J, de los Reyes FL (2021). The role of emptying services in provision of safely managed sanitation: A classification and quantification of the needs of LMICs. J. Environ. Manag..

[64] Oza HH (2022). Occupational health outcomes among sanitation workers: a systematic review and meta-analysis. Int. J. Hyg. Environ. Health.

[65] World Bank, ILO, WHO, & WaterAid. *Health, Safety and Dignity of Sanitation Workers*. 10.1596/32640 (2019).

[66] Lawrence, A. R. & Macdonald, D. M. J. *Guidelines for Assessing the Risk to Groundwater from On-Site Sanitation*. https://nora.nerc.ac.uk/id/eprint/20757/1/ARGOSS%20Manual.PDF (2001).

[67] Still, D. A. & Foxon, K. M. *How Fast Do Pit Toilets Fill up? A Scientific Understanding of Sludge Build up and Accumulation in Pit Latrines* Vol. 2 (Water Research Commission, Gezina, 2012).

[68] Still, D. A., Salisbury, R. H., Foxon, K. M., Buckley, C. A. & Bhagwan, J. N. The challenges of dealing with full VIP latrines. *Proc. WISA Bienn. Conf. Exhib. Durb. ICC South Afr*. 18–22 (2005).

[69] Nakagiri A (2015). Are pit latrines in urban areas of Sub-Saharan Africa performing? A review of usage, filling, insects and odour nuisances. BMC Public Health.

[70] Prasad P (2021). Methods for estimating quantities and qualities (Q&Q) of faecal sludge: field evaluation in Sircilla, India. J. Water Sanit. Hyg. Dev..

[71] Moonkawin J (2023). Challenges to accurate estimation of methane emission from septic tanks with long emptying intervals. Environ. Sci. Technol..

[72] Norris, J. *Sludge Build-Up in Septic Tanks, Biological Digesters and Pit Latrines in South Africa*. https://www.wrc.org.za/wp-content/uploads/mdocs/544-1-00.pdf (2000).

[73] UNICEF. MICS7 Base Household Questionnaire 7.1.8. New York: United Nations Children’s Fund (UNICEF). https://mics.unicef.org/tools?round=mics7 (2023).

