# Supplementary material for: Indicators to complement global monitoring of safely managed on-site sanitation to understand health risks
Source: NPJ Clean Water. 2024 Jul 7;7(1):58. doi: 10.1038/s41545-024-00353-2 (PMC11227438; doi:10.1038/s41545-024-00353-2)
Supplement: Supplementary file 1 — Supplementary Materials [file 41545_2024_353_MOESM1_ESM.pdf]

Indicators to complement global monitoring of safely managed on-site sanitation to understand health risks: Supplementary information

Descriptive results of cities and districts

Supplementary Table 1. Summary of city characteristics

			Background characteristics from survey results			
**Country and city**	**Household sample size**	**City population[a]**	**% on-site sanitation[b]**	**Average depth groundwater**	**% drinking supply from groundwater**	**Main soil type[c]**
**URBAN CITIES**	**26,436**					
**Bangladesh**	**11,995**	(2022)				
Benapole	1270	36,524		3-5m	95%	Clay
Gazipur	463	5,433,563	100%	>20m	5%	Clay
Jessore	1543	3,147,039	100%	5-10m	96%	Clay
Jhenaidah	1872	2,051,607	100%	5-10m	68%	Gravel and sand
Khulna	2912	2,673,002	100%	5-10m	52%	Peat and clay
Kushtia	2703	2,198,731	100%	3-5m	39%	Clay
Tongi	1232	350,000 (2011)	100%	>20m	2%	Clay
**Indonesia**	**5,038**	(2020)				
Bandar Lampung	2413	1,166,066	100%	10-20m	29%	Gravel, clay, peat
Metro	1069	168,676	100%	5-10m	74%	Clay
Tasikmalaya	1556	716,155	100%	3-5m	49%	Gravel and sand
**Nepal**	**2,960**	(2021)				
Birendranagar	1087	153,863	100%	5-20m	16%	Clay
Chandannath	393	21,036	100%	>20m	0%	Clay
Khadak	392	52,778	100%	5-20m	98%	Clay
Nepalgunj	1088	164,444	100%	2-3m	92%	Fine sand
**Tanzania**	**3,613**	(2022)				
Arusha	2507	617,631	97%	1-3m	5%	Clay
Shinyanga	1106	139,727	100%	2-5m	3%	Clay
**Zambia**	**2,830**	(2010 - district)				
Kabwe	1121	202,360	66%	1-3m	46%	Fine sand and gravel
Kasama	636	231,824	96%	10-20m	25%	Clay and gravel
Mbala	271	203,129	95%	2-20m	18%	Clay
Mpulungu	395	98,073	99%	3-5m	46%	Gravel, sand and fractured rock
Nakonde	407	119,708	100%	5-10m	86%	Clay
**RURAL DISTRICTS**	**5,348**					
**Bhutan**	**2,620**	(2017)				
Chhukha	464	68,966	100%	>10m	11%	Clay
Dagana	303	24,965			1%	
Lhuentse	137	14,437			0%	
Pemagatshel	177	23,632			0%	
Punakha	362	28,740			0%	
Samtse	598	62,590			2%	
Trashigang	386	45,518			0%	
Zhemgang	193	17,763			1%	
**Laos**	**1,945**	(2015)				
Atsaphone	552	59,580	100%	1- >10m	82%	Clay
Champhone	994	109,174		1-10m	17%	Sand, gravel, clay
Phalanxay	399	40,097		1-5m	51%	Gravel
**Nepal**	**783**	(2021)				
Dailekh	315	252,313	100%	>10m	10%	Clay
Sarlahi	468	862,470		2-3m	100%	Clay

a. Population from http://www.citypopulation.de/
b. % of surveyed population using on-site sanitation (septic tanks and latrines) of improved sanitation (on-site and sewer).
c. Soil type: Clay = Heavy clay/loam, Gravel = gravel or course sand,

Summary of sanitation data

Summary of the country data for the global indicator ladder of sanitation. Please note that while the safely managed sanitation estimate for the global monitoring is only based on those that do not share their facilities (at least basic) the analysis in the paper was based on improved OSS, as it was for the purpose of comparison of impact rather than global reporting against SDG.

Supplementary Table 2. Sanitation ladder based on global indicators and showing pit type.

	Urban					Rural			Average all countries
	Bangladesh	Indonesia	Nepal	Tanzania	Zambia	Bhutan	Laos	Nepal	
**Open Defecation**	0%	4%	7%	3%	3%	4%	52%	4%	10%
**Unimproved**	11%	19%	1%	9%	18%	9%	0%	0%	8%
**Improved (Sewer)**	0%	0%	0%	2%	10%	0%	0%	0%	1%
**Improved (OSS)**	89%	77%	93%	87%	68%	87%	48%	96%	81%
- Improved and contained OSS	37%	63%	87%	84%	67%	82%	45%	92%	70%
- Improved and emptied OSS	36%	8%	14%	11%	2%	2%	10%	11%	12%
**Limited**	22%	5%	15%	29%	18%	8%	5%	4%	13%
**Basic Sewer**	0%	0%	0%	1%	10%	0%	0%	0%	1%
**Basic OSS**	67%	72%	78%	59%	51%	79%	42%	92%	68%
- Basic and contained	29%	59%	72%	57%	50%	79%	42%	91%	60%
- Basic, contained and emptied	10%	5%	9%	5%	1%	2%	4%	10%	6%
- Basic contained and stored in-situ	19%	54%	64%	48%	49%	77%	38%	82%	54%
**Containment of improved OSS** (% respondents)									
Uncontained OSS	52%	17%	11%	7%	5%	0%	3%	8%	14%
Contained OSS	31%	60%	81%	79%	63%	81%	42%	85%	66%
**Emptying of improved OSS** (% respondents)									
Previously emptied	32%	6%	13%	10%	1%	2%	4%	10%	10%
Never emptied or don't know	55%	69%	79%	66%	63%	80%	40%	83%	67%
Built a new pit	1%	2%	1%	5%	1%	0%	0%	0%	1%

Supplementary Table 3. Type of improved sanitation

Type of improved sanitation	Urban					Rural			Average all countries
	Bangladesh	Indonesia	Nepal	Tanzania	Zambia	Bhutan	Laos	Nepal	
**Direct pit**	6%	4%	2%	32%	57%	12%	9%	7%	16%
Off-set pit	22%	10%	51%	40%	6%	71%	29%	84%	39%
Double (alternating) off-set pit	5%	0%	4%	2%	1%	1%	53%	0%	8%
Two (or more) sequential pits	15%	0%	1%	0%	0%	0%	7%	1%	3%
Single compartment (for composting and UDTs)	0%	0%	0%	3%	1%	0%	0%	0%	0%
Water tight tank	3%	1%	29%	2%	0%	16%	1%	7%	7%
Septic tank	47%	79%	13%	18%	21%	0%	0%	1%	23%
Communal septic tank	1%	6%	0%	1%	1%	0%	0%	0%	1%
Piped sewer or DEWATS	0%	0%	0%	2%	13%	0%	0%	0%	2%

Supplementary Table 4. Key context variables for each country for improved on-site sanitation systems

	Urban					Rural		
	Bangladesh	Indonesia	Nepal	Tanzania	Zambia	Bhutan	Laos	Nepal
% Poorer households (Lowest two wealth quintiles)	32%	6%	6%	1%	35%	35%	7%	29%
% GW depth less than 5m	34%	19%	39%	92%	41%	0%	67%	62%
% Dry containment (not water-based/wet)	5%	0%	1%	17%	62%	8%	1%	1%
% Pit (not tank)	49%	14%	59%	79%	74%	84%	98%	92%
% Age toilet more than 5 years	67%	77%	60%	47%	28%	48%	58%	39%
% Depth containment less than 3m	4%	31%	59%	1%	23%	57%	35%	27%

Supplementary Table 5. Comparison of average pit/tank depth between cities

	% OSS with depth estimate	Depth of tank/pit raw data				% High confidence in self-reported	Infiltration depth (Groundwater level – containment depth)	
		Average	Min	Max	St Dev		Average	St Dev
Bangladesh	89%	8.2	0.5	45	4.9	26%	2.8	6.6
Indonesia	92%	3.0	1.0	200	4.7	46%	9.8	6.1
Nepal	99%	2.4	1.0	4	0.6	65%	10.6	10.1
Tanzania	95%	12.0	2.0	83	7.5	46%	-4.9	3.6
Zambia	80%	3.3	1.0	20	1.9	28%	7.9	9.7
Bhutan	100%	2.39	1	20	0.6	NA	17.6	0.6
Laos	100%	2.11	1	10	0.7	NA	4.3	5.3
Nepal	100%	2.89	1	5	0.8	NA	6.6	8.6

Supplementary Table 6. Urban timely emptying threshold per country and containment type

		Bangladesh		Indonesia		Nepal		Tanzania		Zambia	
	**HH members**	4.8		4		6		5		4.1	
**Type**	Accumulation rate (m3 /cap / year)	Final size (m3)	Thres -hold Years	Final size (m3)	Thres -hold Years	Final size (m3)	Thres -hold Years	Final size (m3)	Thres -hold Years	Final size (m3)	Thres-hold Years
Pit (direct or off-set)	0.06	1.57	5.5	1.57	6.5	1.57	4.4	2.36	7.9	5.6	22.8
Twin pits (sequential)	0.06	3.14	10.9	3.14	13.1	3.14	8.7	4.72	15.7	11.2	45.5
Twin pits (alternating)	0.06	1.57	5.5	1.57	6.5	1.57	4.4	2.36	7.9	5.6	22.8
Dry / composting (single compartment)	0.04	0.12	0.6	0.12	0.8	0.12	0.5	0.12	0.6	0.12	0.7
Dry / composting (double compartment)	0.04	0.24	1.3	0.24	1.5	0.24	1.0	0.24	1.2	0.24	1.5
Septic tank, holding tank	0.08	7.5	19.5	1.8	5.6	7.5	15.6	1.26	3.2	8.3	25.3

Supplementary Table 7. Rural timely emptying threshold per country and containment type

		Bhutan		Laos		Nepal	
	**HH members**	4		5		6	
**Type**	Accumulation rate (m3/cap/year)	Final size (m3)	Threshold Years	Final size (m3)	Threshold Years	Final size (m3)	Threshold Years
Pit (direct or off-set)	0.06	2.10	8.8	1.26	4.2	1.57	4.4
Twin pits (sequential)	0.06	4.21	17.5	2.51	8.4	3.14	8.7
Twin pits (alternating)	0.06	2.10	8.8	2.51*	8.4*	1.57	4.4
Dry / composting (single compartment)	0.04	0.12	0.8	0.12	0.6	0.12	0.5
Dry / composting (double compartment)	0.04	0.24	1.5	0.24	1.2	0.24	1.0
Septic tank, holding tank	0.08	7.5	19.5	6	15.0	7.5	15.6

* Note that Laos considered the volume of alternating pits equivalent to sequential pits which differs from other countries.

Complementary indicator further analysis by country

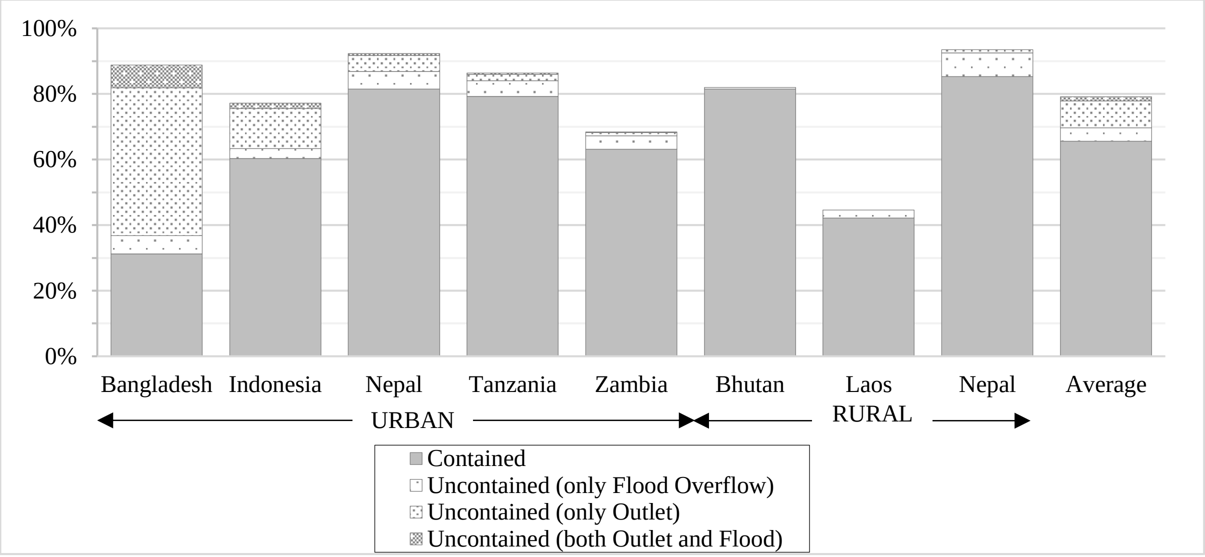

Supplementary Figure 1. Assessment of the global indicator for containment highlighting the fraction of uncontained due to outlets vs. overflow. Outlets to surface environment were a major containment risk in Bangladesh and Indonesia, while overflow was common across countries.

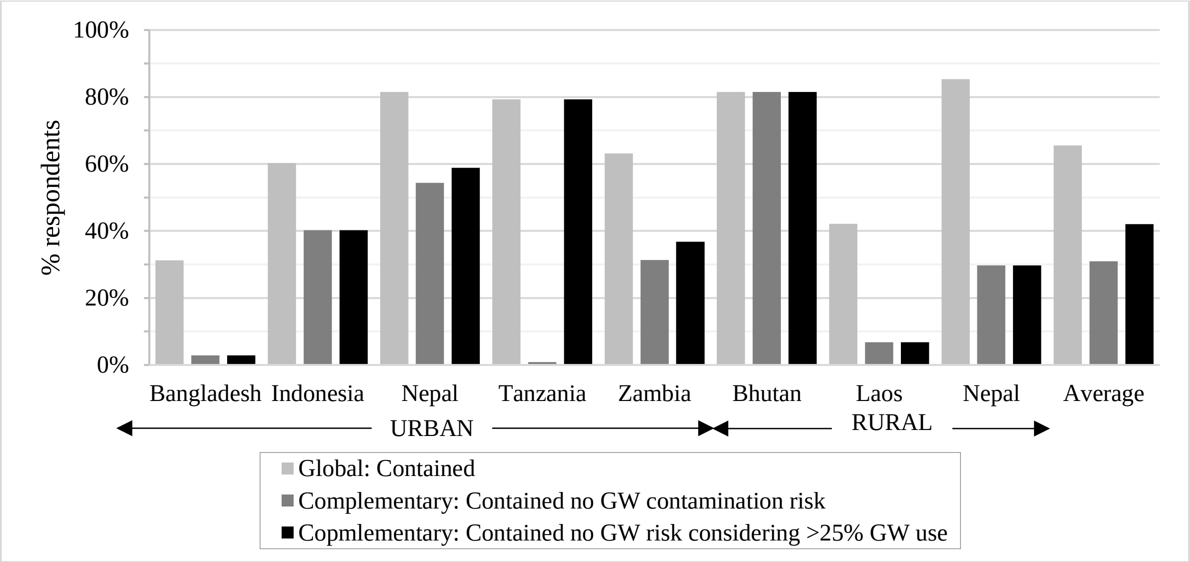

Supplementary Figure 2. Comparison of global indicator for containment, the proposed complementary indicator of groundwater contamination risk and an alternative complementary indicator of contamination risk only where groundwater use was reported by more than 25% of the population. The alternative indicator demonstrates containments in Tanzania pose a risk of groundwater contamination, but this is less of a health risk due to groundwater not being used for drinking.

Supplementary Table 8. Variation in the impact of complementary indicators between and within countries

	% respondents (country average)		Between countries variation in difference between global and complementary indicators		Within-country variation (difference in global and complementary indicators for each city/district)	
	Global	Global + Complementary	St. Dev	Range of differences (min to max)	Average of country St Dev (range of St Dev)	Range of city/district differences showing examples of largest and smallest range
**Animals access**	81%	66%	12%	1-29%	6% (2-13%)	14-51% in Bhutan, 0-2% in Laos
**Groundwater risk**	66%	31%	24%	0-78%	23% (0%-66%)	0-93% rural Nepal, 0% Bhutan
**Overdue for emptying**	67%	45%	10%	8-42%	11% (4-17%)	11-44% urban Nepal, 14-25% Tanzania
**Entered to empty**	10%	8%	2%	0-5%	0.5% (0-1.1%)	2-5% Bangladesh, 0-0.5% Zambia
**Inadequate PPE to empty**	10%	3%	9%	1-29%	4% (0-11%)	11-43% Bangladesh, 0-1% Zambia

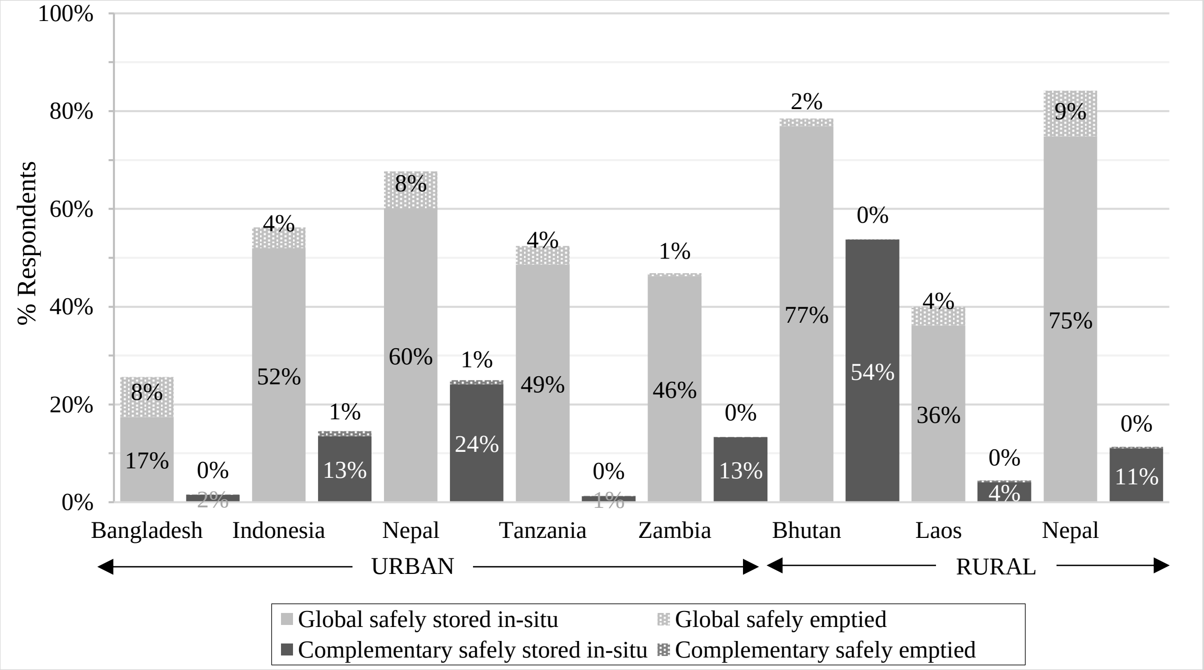

Supplementary Figure 3. Country comparison of the cumulative estimate of safely managed on-site sanitation across the service chain (excluding transport and treatment) for the global and complementary indicators disaggregated by those stored in-situ (safely managed) and those emptied (potentially safely managed).

Complementary indicators analysis at a city or district level

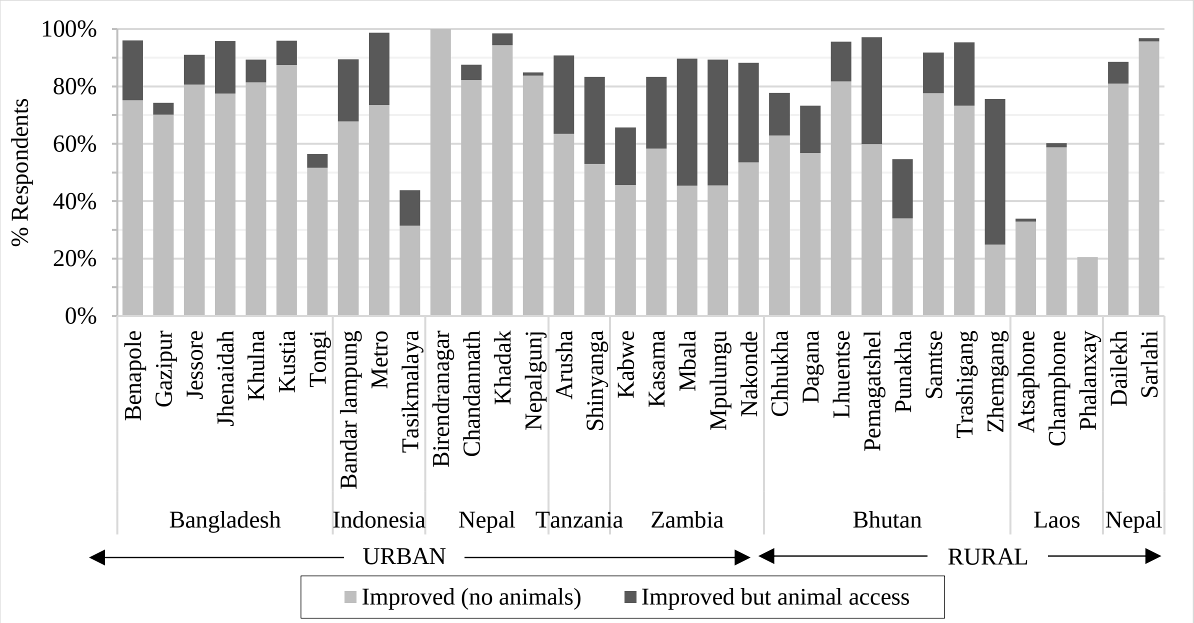

Supplementary Figure 4. Comparison of global indicator for improved sanitation and complementary indicator for no animal access to improved sanitation facilities by city or district. Light grey shows the complementary indicator of improved sanitation without animal access, with animal access to improved sanitation facilities shown in dark. The total column height is equivalent to the global indicator for improved sanitation.

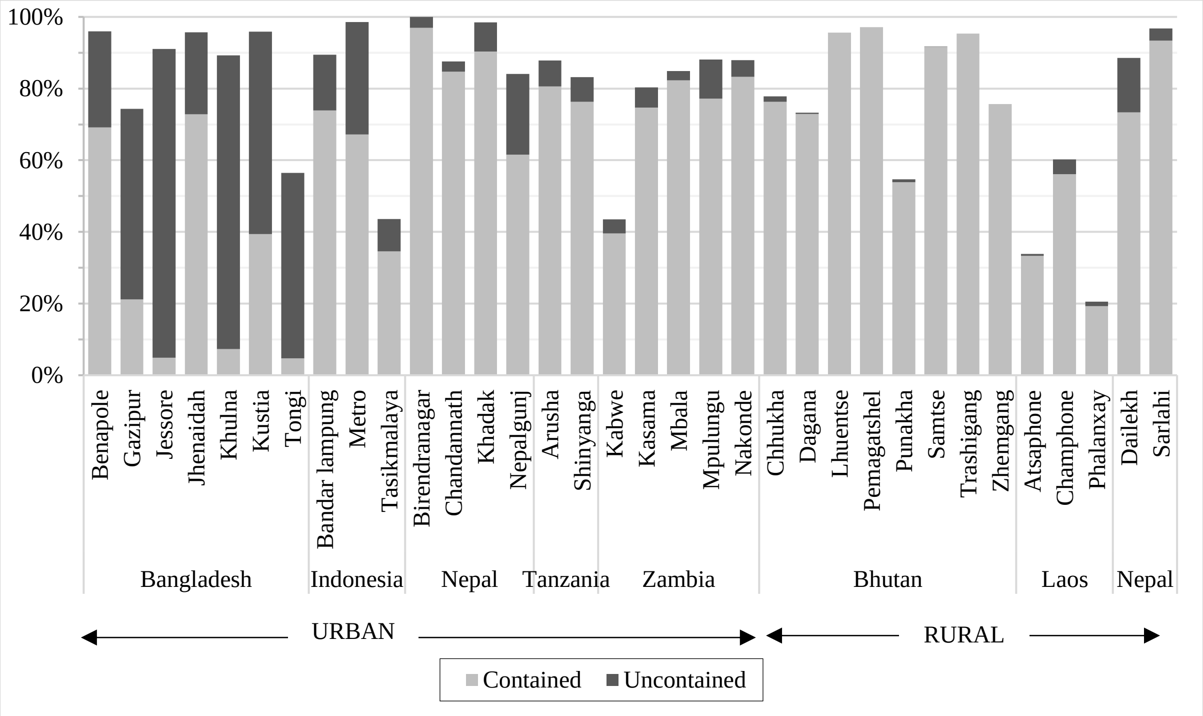

Supplementary Figure 5. Global indicator for contained on-site sanitation demonstrating the proportion contained in light grey and the uncontained in dark grey (outlet to surface environment and/or flooding and overflow). The total column height equals the proportion of respondents with improved on-site sanitation.

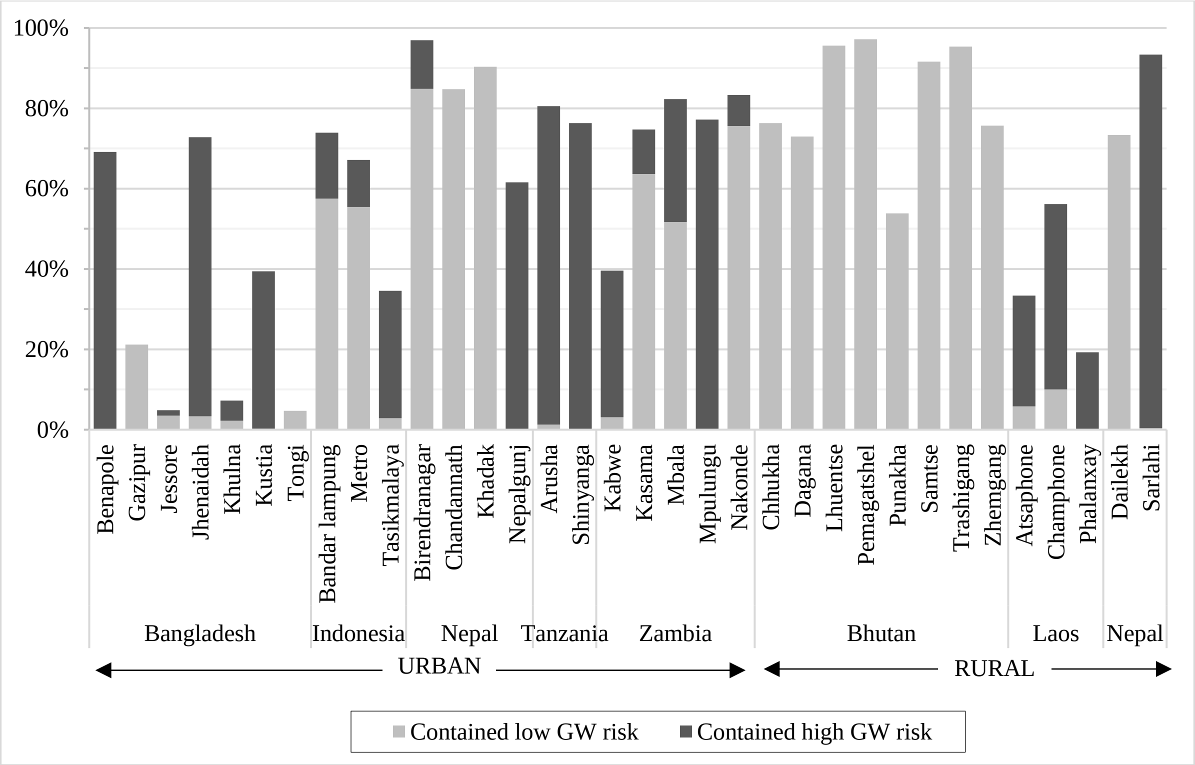

Supplementary Figure 6. Comparison of global indicator for contained on-site sanitation and the complementary indicator for a contained system with a low risk of groundwater contamination by city and district. Light grey shows the complementary indicator of contained sanitation with a low risk of groundwater contamination. High contamination risk is shown in dark. The total column height is equivalent to the global indicator for contained sanitation.

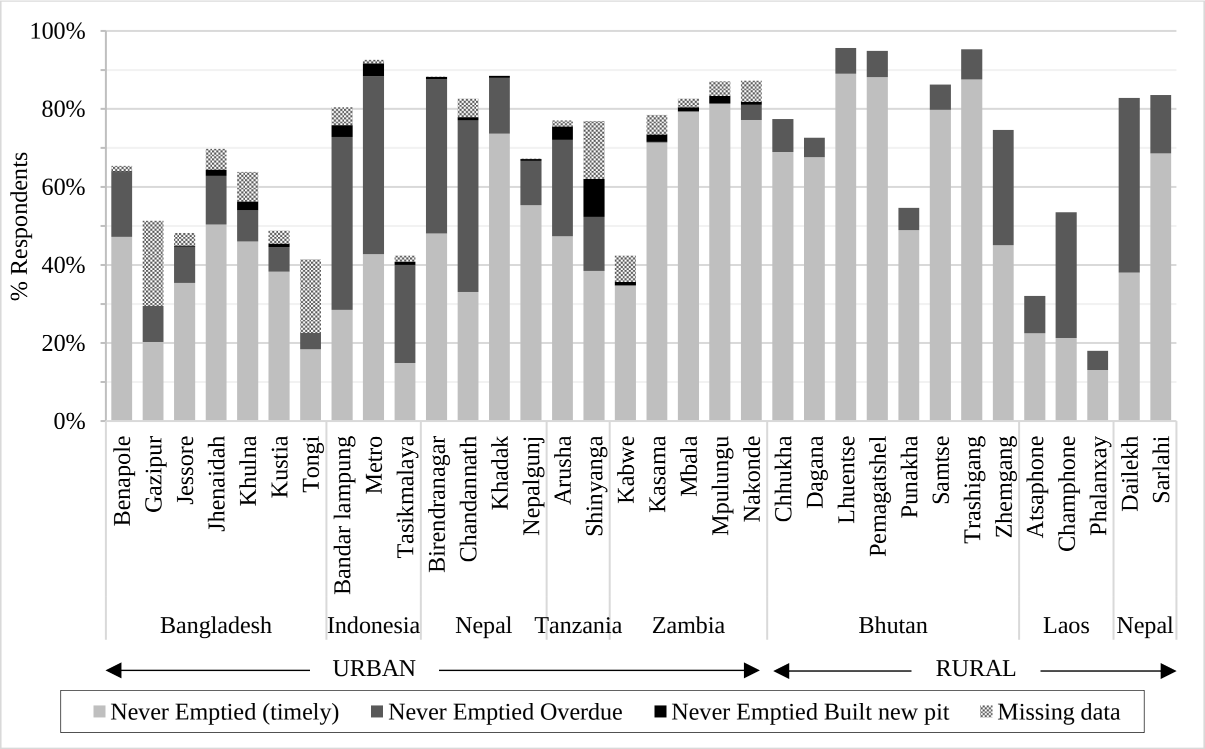

Supplementary Figure 7. Comparison of the global indicator for never emptied (safely stored in-situ) and the complementary indicator considering the timely emptying threshold. Light grey shows the complementary indicator of improved never emptied OSS that are below the emptying threshold, with the dark grey those overdue for emptying. Black indicates the respondents reporting they had never emptied but built a new pit and the pattern is missing data on emptying or age of operation.

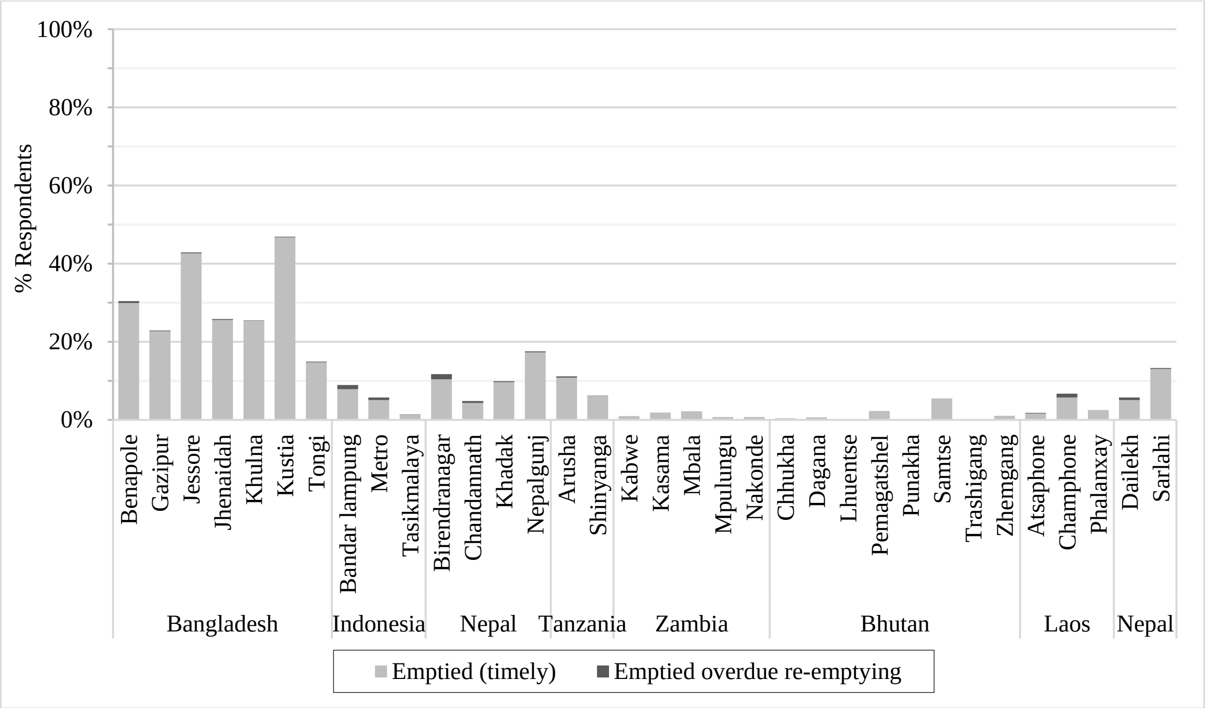

Supplementary Figure 8. Comparison of global and complementary indicator for timely emptying of emptied containments. Light grey shows the complementary indicator of improved, emptied on-site sanitation not exceeding the timely threshold, with the systems overdue for re-emptying shown in dark. The total column height is equivalent to the global indicator for emptied sanitation.

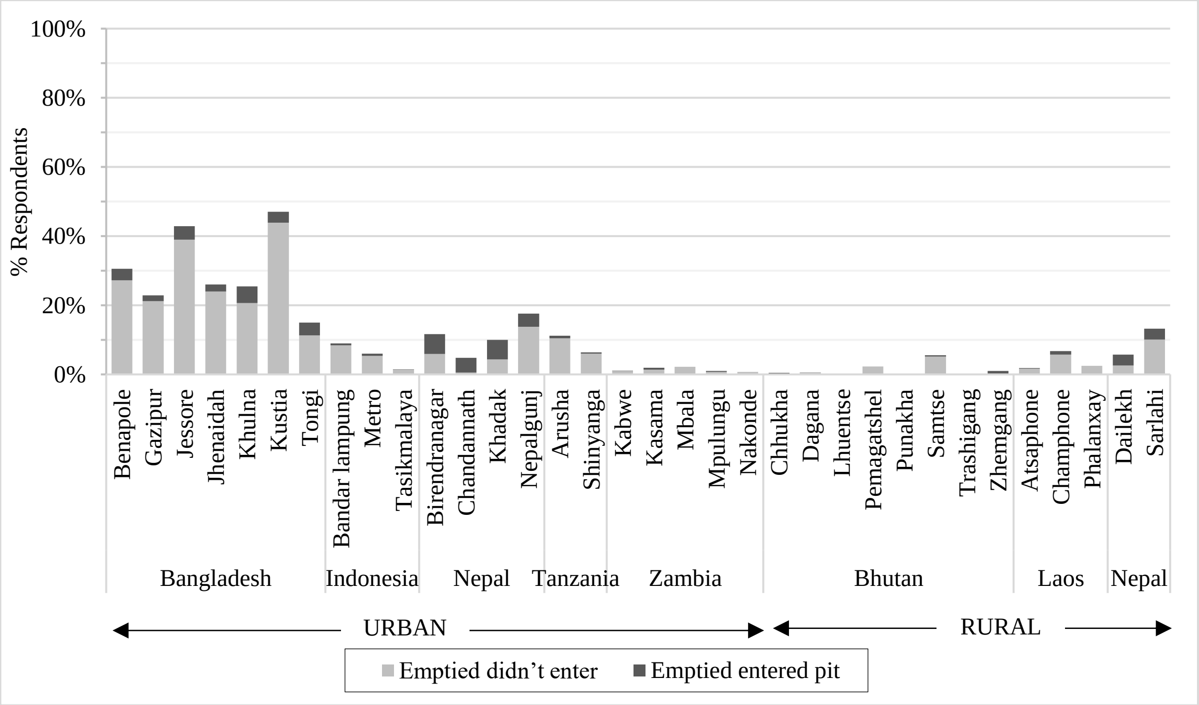

Supplementary Figure 9. Comparison of the global indicator for emptying improved on-site sanitation and the complementary indicator for emptying without entering the containment by city or district. Light grey shows the complementary indicator of emptied containments that were not entered, with the systems entered to empty shown in dark. The total column height is equivalent to the global indicator for emptied sanitation.

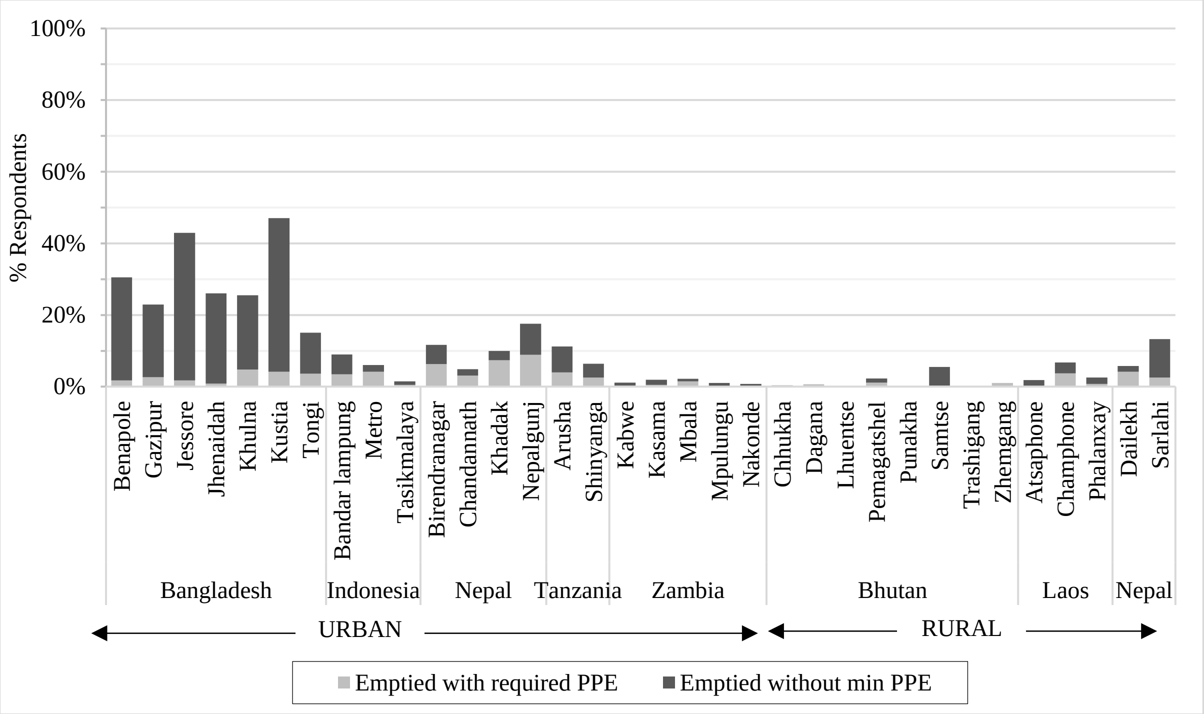

Supplementary Figure 10. Comparison of the global indicator for emptying improved on-site sanitation facilities and complementary indicator on emptying with adequate PPE by city or district. Light grey shows the complementary indicator of emptied containments that reported adequate use of PPE, with the systems without adequate PPE shown in dark. The total column height is equivalent to the global indicator for emptied sanitation.

Questionnaire used in the urban baseline data collection

Supplementary Table 9. Detailed questions and responses from SNV urban sanitation survey used in this analysis

Indicator	Question	Responses
**Overarching questions**	Do members of your household have a toilet?	No toilet, practice OD<br>No own toilet, use of shared toilet (or neighbour's)<br>Use of communal toilet<br>Use of one cubicle in a communal toilet block<br>Use of own household toilet
	Ask and observe question: What type of toilet is it? Can you please show it to me?<br>(*only answer if above indicates use of a toilet)*	Pour flush toilet<br>Cistern flush toilet<br>Ventilated improved pit latrine (VIP)<br>Pit latrine with slab<br>Pit latrine without slab<br>Composting toilet<br>Urine diversion toilet (UDT)<br>Bucket<br>Hanging toilet or hanging latrine
	Ask and observe question: Where do the faeces go?<br>(*Only answer if above was an improve toilet or pit latrine without slab)*	To the street- field or open pit<br>To a pond<br>To the river, waterway or open drain<br>To a closed drain<br>To a direct pit<br>To an off-set pit<br>To a double (alternating) off-set pit<br>To two (or more) sequential pits<br>To a single compartment (for composting and UDTs)<br>To a double compartment (for composting and UDTs)<br>To a water tight tank<br>To a septic tank<br>To a communal septic tank<br>To piped sewer or DEWATS
	Ask and observe question: Is there an effluent outlet?<br>(*Only answer if above response was a containment)*	Yes<br>No
	Ask and observe question: Where does the effluent go?<br>(*Only answer if you responded Yes to above)*	To the street or open field<br>To an uncovered drain<br>To a covered drain<br>To a water stream

Indicator	Question	Responses	
		To soak pit or soak well<br>To sewer or other piped system	
**Animal access to excreta:** Rats and flies cannot enter and exit the toilet or containment	Can rats access the faeces in any way?<br>*Only answer if have or use a toilet*	Yes<br>No	
	Does the toilet pan or slab allows flies to enter and exit the pit? *(Note only asked to those that responded no to the first one, as access to rats means flies could also enter)*	Yes<br>No	
**Flooding and overflow*:** Pit or tank does not flood, overflow or leak	Does the toilet flood at any time of the year?<br>(*Note this was not included in rural surveys*)	Yes<br>No	
	Does the pit or toilet leak, overflow or flood at any time of the year?	Yes, sewage backflows into the toilet or property<br>Yes, the pit is leaking into the property<br>Yes, the pit is overflowing or impossible to flush<br>No, sewer works well<br>No, pit or tank works well<br>Don't know	
	How often does it leak or overflow? *(Note only asked to those that responded yes to leak, overflow or flood)*	It happened only once<br>When there is a very heavy rain<br>Regularly during the rainy season<br>Continuously<br>Don't know	
**Groundwater risk:** Low risk to groundwater from subsurface leaching of pits or tanks	How deep is the toilet pit below the surface? *(Note this is asked to all pits/tanks, indicating depth should be from surface to base on pit or tank.)*	m	
	What is the main source for drinking water?	Piped into dwelling<br>Piped to yard/plot<br>Communal tap<br>Tanker truck<br>Cart with small tank<br>River/stream<br>Pond/lake/dam<br>Rainwater<br>Surface water (river/dam/ lake/pond/stream/canal/ irrigation channel)<br>Bottled water	Tube well or borehole<br>Protected well<br>Unprotected well<br>Protected spring<br>Unprotected spring<br>Protected public well/borehole<br>Spring
	Local government question: What is the predominate soil type in the neighbourhood / sub-district?	Solid rock<br>Peat<br>Heavy clay/loam	Fine sand<br>Gravel or coarse sand<br>Fractured rock
	Local government question: What is the typical depth of groundwater in the neighbourhood / sub-district?	Less than 1 metre<br>Between 2-3 metres<br>Between 3-5 metres	Between 5-10 metres<br>Between 10-20 metres<br>More than 20 metres
**Not emptied OSS within timely threshold:** Never emptied pits or tanks, age below timely emptying threshold	How old is your toilet (pit/tank)?	Less than 1 year<br>1-3 years<br>4-5 years<br>Older than 5 years<br>Don't know	
	Has the pit or tank ever been emptied?	Yes<br>No- the pit is not full yet<br>No- we have already dug a new pit<br>No, it is a sewer connection<br>Don't know	
**Emptied OSS within timely threshold:** Years since pits or tanks were emptied within timely emptying threshold	When was the last time the pit or tank was emptied? (if emptied)	Less than 6 months ago<br>6- 12 months ago<br>1-3 years ago<br>4-10 years ago<br>More than 10 years ago<br>Don't know	
	Do you share this toilet with people who are not a member of your household?	No, only used by own household<br>Yes, with neighbour's household<br>Yes, with more than two households	
**Emptying health and safety risks:** Emptying of containments does not pose a health and safety risk to workers or the public	To empty the pit, did someone need to enter the pit?	Yes<br>No<br>Don't know	
	Did you observe any of the following safter measures during emptying? (use of boots, gloves and a mask)	Workers were wearing boots and gloves<br>Workers were wearing face masks<br>Workers cleaned up any spills before they left<br>Workers washed hands with water and soap before they left<br>Vehicle was well closed and not leaking<br>None of the above<br>Don't know	
